# Proteolytic cleavage activates the mitochondrial isoform of TOP3A

**DOI:** 10.1093/nar/gkaf1140

**Published:** 2025-11-04

**Authors:** Direnis Erdinc, Christin A Albus, Alejandro Rodríguez-Luis, Katja E Menger, Annika Thorsell, Ilian Atanassov, Urška Rovšnik, Maria Falkenberg, Claes M Gustafsson, Thomas J J Nicholls

**Affiliations:** Department of Medical Biochemistry and Cell Biology, University of Gothenburg, SE-405 30 Gothenburg, Sweden; Biosciences Institute, Faculty of Medical Sciences, Newcastle University, Newcastle upon Tyne NE2 4HH, United Kingdom; Biosciences Institute, Faculty of Medical Sciences, Newcastle University, Newcastle upon Tyne NE2 4HH, United Kingdom; Biosciences Institute, Faculty of Medical Sciences, Newcastle University, Newcastle upon Tyne NE2 4HH, United Kingdom; Proteomics Core Facility at Sahlgrenska Academy, University of Gothenburg, SE-405 30 Gothenburg, Sweden; Proteomics Core Facility, Max-Planck-Institute for Biology of Ageing, 50931 Cologne, Germany; Department of Medical Biochemistry and Cell Biology, University of Gothenburg, SE-405 30 Gothenburg, Sweden; Department of Medical Biochemistry and Cell Biology, University of Gothenburg, SE-405 30 Gothenburg, Sweden; Department of Medical Biochemistry and Cell Biology, University of Gothenburg, SE-405 30 Gothenburg, Sweden; Biosciences Institute, Faculty of Medical Sciences, Newcastle University, Newcastle upon Tyne NE2 4HH, United Kingdom

## Abstract

The TOP3A gene encodes two isoforms, one targeted to the nucleus and one to mitochondria. Nuclear TOP3A functions as part of the BTRR complex to resolve double Holliday junctions during homologous recombination, while the mitochondrial isoform separates hemicatenated daughter mitochondrial DNA (mtDNA) molecules following DNA replication. Here, we show that the mitochondrial isoform of TOP3A undergoes proteolytic cleavage by the mitochondrial processing peptidase, removing ~90 amino acids from the C-terminus. This cleavage enhances the enzyme’s biochemical properties, increasing single-stranded DNA binding and decatenation activity. Notably, all BTRR complex subunits, except TOP3A, are absent from mitochondria, suggesting that proteolytic processing enables TOP3A to function autonomously in mtDNA maintenance. We propose that this cleavage represents a post-import maturation step that tailors TOP3A to its mitochondrial context by uncoupling it from nuclear protein interactions and enhancing its catalytic efficiency.

## Graphical Abstract

**Graphical Abstract:**
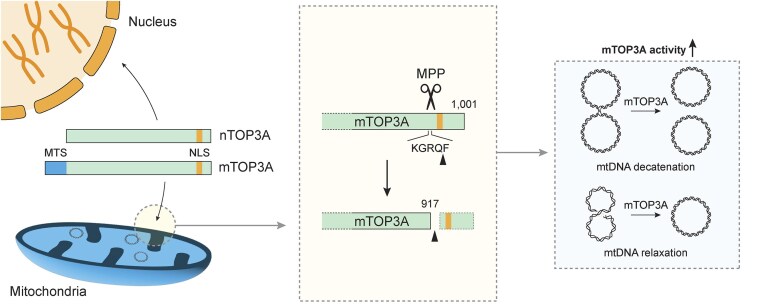

## Introduction

The mitochondria of eukaryotic cells are descended from an ancient merger in which an α-proteobacterium was engulfed by a host cell. Mitochondria contain their own genome, called mitochondrial DNA (mtDNA), which is a highly-reduced remnant of the genome of this bacterial endosymbiont [1]. Of ~1200 proteins that are found in mitochondria [2], only 13 are encoded by mtDNA, all of which are subunits of the oxidative phosphorylation system. This means that all other mitochondrial proteins, including those required for mtDNA replication, expression, and repair, are nuclear-encoded and imported post-translationally into mitochondria via targeting sequences.

The protein machineries required for mtDNA replication and expression are largely distinct from those in the nucleus [3]. Most core proteins required for mtDNA replication and expression function only within mitochondria, and are homologous to either bacteriophage proteins (including the DNA polymerase POLG [4], helicase TWINKLE [5], and RNA polymerase POLRMT [6]) or bacterial proteins (including the single-stranded DNA (ssDNA) binding protein MTSSB [7] and subunits of the mitochondrial ribosome [8]). However, a subset of genes encode protein isoforms targeted to both mitochondria and the nucleus or cytosol, allowing them to function in multiple compartments. This includes the topoisomerase TOP3A [9], the transfer RNA processing enzyme ELAC2 [10], and a number of DNA glycosylases involved in base excision repair [11–13].

TOP3A is an enzyme that alters DNA topology and is critical for processes such as replication and recombination by creating breaks in the DNA backbone. The strand-passage mechanism of TOP3A allows it to remove negative supercoiling, and also to resolve single-stranded interlinks between DNA strands (hemicatenanes) that would otherwise prevent the segregation of replicated molecules [14]. The TOP3A transcript contains two translation initiation sites, producing longer and shorter isoforms [9]. The longer isoform bears a mitochondrial targeting sequence (MTS) at its N-terminus, which targets the protein for import into mitochondria, whereas the shorter isoform lacks the MTS and localizes to the nucleus [9, 15, 16]. The MTS of the mitochondrial isoform has been reported to be proteolytically cleaved at approximately A20, very close to the initiation site for the nuclear isoform at M26 [9], meaning that the two mature isoforms would be expected to be very similar in size.

Nuclear TOP3A forms a complex called BTRR, together with the helicase BLM and the OB-fold proteins RMI1 and RMI2 [17]. This complex is able to dissolve double Holliday junctions that form between sister chromatids during homologous recombination [18, 19]. TOP3A has also recently been found to associate with active replication forks, both in the nucleus of human cells [20] and in *Escherichia coli* [21], indicating that it also contributes to the unlinking of replicated chromosomes. In contrast to the nuclear isoform, the mitochondrial isoform of TOP3A acts independently of the other components of BTRR [16]. The loss of mitochondrial TOP3A results in the accumulation of hemicatenated mtDNA replication products centred around the replication origin OriH, and also causes genome-wide replication stalling [15, 16]. This has indicated that the OriH region is a primary site of mtDNA replication termination and that TOP3A normally removes hemicatenanes between replicated daughter mtDNA molecules to allow their segregation [22]. The specialization of TOP3A isoforms is also highlighted by mutations in TOP3A, which can have deleterious effects upon both mtDNA and the nuclear genome. Pathological TOP3A mutations have been associated with two distinct disease phenotypes. The first is an adult-onset mitochondrial disease characterized by progressive external ophthalmoplegia, cerebellar ataxia, cardiac conduction defects, myopathy, and sensory axonal motor neuropathy [16, 23–25]. The second resembles Bloom syndrome but includes additional features of mitochondrial dysfunction [26, 27].

Given the separate roles of nuclear and mitochondrial TOP3A isoforms, and the complexity of TOP3A-related disease, we have investigated factors that may enable TOP3A to functionally adapt to the mitochondrial or nuclear compartments. We find that the mitochondrial isoform of TOP3A specifically undergoes proteolytic processing by the mitochondrial processing peptidase (MPP), removing ~90 amino acids from the C-terminus of the protein. This processing alters the biochemical activity of TOP3A, favouring its ability to decatenate ssDNA replication products. We propose that the proteolytic cleavage of TOP3A represents a functional adaptation to the unique DNA structures encountered in mitochondria.

## Materials and methods

### Cell culture and transfection

Parental Flp-In T-REx 293 and U2OS cells were cultured in Dulbecco’s Modified Eagle Medium (DMEM, Gibco), supplemented with 10% foetal bovine serum (FBS), 100 U/ml penicillin, 100 μg/ml streptomycin, 15 μg/ml blasticidin, and 100 μg/ml Zeocin. Following stable transfection, these cells were maintained in DMEM containing 10% FBS, 100 U/ml penicillin, 100 μg/ml streptomycin, 15 μg/ml blasticidin, and 50 μg/ml hygromycin. For stable transfection, 250 000 cells were plated in antibiotic-free medium the day before transfection. Cells were then transfected using Lipofectamine 3000 according to the manufacturer’s instructions, with 2250 ng of pOG44 and 250 ng of pcDNA5 FRT/TO containing the TOP3A complementary DNA of interest cloned in frame with BirA*-HA. Cells were transferred to 9-cm dishes 24 h after transfection, and then 24 h later the culture medium was replaced with selective medium containing 15 μg/ml blasticidin and 50 μg/ml hygromycin. Antibiotic-resistant colonies were isolated after 2–3 weeks using glass cloning cylinders. For cell fractionation experiments, the expression of TOP3A constructs was induced in stably transfected Flp-In T-REx 293 by treatment with 100 ng/ml doxycycline for 48 h.

### Molecular cloning and mutagenesis

For transfection of human cells, the TOP3A coding sequence (Genscript NM 004618.5) was amplified without the stop codon, incorporating 5′ KpnI and 3′ XhoI restriction sites. The polymerase chain reaction product was then cloned into pcDNA5/FRT/TO in frame with BirA*-HA, which had been inserted between the XhoI and ApaI restriction sites.

Variants of TOP3A used for alanine scanning experiments, as well as M1L variants, were generated using the QuikChange Lightning Site-Directed Mutagenesis Kit (Agilent Technologies) according to the manufacturer’s instructions. Successful mutagenesis was confirmed by Sanger sequencing (Eurofins MWG Operon).

For protein purification, the human TOP3A coding sequence (amino acids 16-1001, 16-934, and 16-917, with an N-terminal TEV-cleavable 6 × His-tag) was cloned into the pBacPAK9 vector (Clontech) using 5′ SacI and 3′ XhoI restriction sites.

### Sodium dodecyl sulphate–polyacrylamide gel electrophoresis and western blotting

For visualization of recombinant proteins, samples were separated on a 4%–20% stain-free Criterion TGX sodium dodecyl sulphate–polyacrylamide gel electrophoresis (SDS–PAGE) gel (Bio-Rad) and imaged using a Stain-Free imager (Bio-Rad). For western blotting, sample pellets were solubilized in lysis buffer [50 mM Tris–HCl, pH 7.4, 150 mM NaCl, 1 mM ethylenediaminetetraacetic acid (EDTA), 1% (v/v) Triton X-100, and 1 × protease inhibitors (Thermo Scientific A32965)], incubated on a roller at 4°C for 30 min, then centrifuged at 11 000 × *g* for 3 min at 4°C. The supernatant was retained and used for protein quantification using a BCA assay (Pierce). Equal protein quantities were separated on 4%–20% Criterion TGX SDS–PAGE gels (Bio-Rad), electroblotted onto nitrocellulose membrane, and blocked using 5% (w/v) milk (Marvel) in phosphate buffered saline (PBS) for 1 h at room temperature. Primary antibodies were then added and incubated overnight on a roller at 4°C. Membranes were washed for 3 × 10 min with PBS containing 0.1% (v/v) Triton X-100, incubated with secondary antibodies for 1 h at room temperature in 5% (w/v) milk in PBS, washed a further 3 × 10 min with PBS containing 0.1% (v/v) Triton X-100, then developed using Pierce ECL (Thermo Scientific 32109), SuperSignal West Pico PLUS (Thermo Scientific 34580), or SuperSignal West Femto (Thermo Scientific 34096). Antibody details are provided in Supplementary Table S1.

### Subcellular fractionation

Mitochondrial isolation was carried out as described in [28]. Cells were suspended in nine volumes (assuming a cell density of 1.25 g/ml) of hypotonic buffer [20 mM HEPES, pH 8, 5 mM KCl, 1.5 mM MgCl_2_, 2 mM dithiothreitol (DTT), 1 mg/ml bovine serum albumin (BSA), 1 mM phenylmethylsulfonyl fluoride (PMSF), and 1 × protease inhibitors] and incubated on ice for 10 min. Cells were homogenized using 10 strokes of a 15 ml glass Dounce homogenizer, then a two-thirds volume was added of 2.5 × MSH (525 mM mannitol, 175 mM sucrose, 20 mM HEPES, pH 8, 5 mM EDTA, 1 mg/ml BSA, 0.2 mM PMSF, 2 mM DTT, and 1 × protease inhibitors), and a sample retained as the whole cell extract (WCE) fraction. This homogenate was centrifuged twice for 10 min at 1600 × *g* at 4°C, with the pellet from this first centrifugation step retained as the nuclear fraction, and then the supernatant was centrifuged again at 10 000 × *g* for 10 min at 4°C. The supernatant from this step was retained as the cytosolic fraction. The pellet was resuspended in DNase buffer (210 mM mannitol, 70 mM sucrose, 20 mM HEPES, pH 8, 10 mM MgCl_2_, 2 mM EDTA, 1 mg/ml BSA, 1 mM PMSF, and 1 × protease inhibitors), treated with 0.2 mg/ml DNase I (Sigma–Aldrich DN25) on a roller for 1 h at 4°C, then EDTA was added to a final concentration of 15 mM, and the sample centrifuged at 10 000 × *g* for 10 min at 4°C. The pellet was washed twice with 1 × MSH (DNase buffer without BSA or MgCl_2_), loaded onto a two-step 1.5 M/1 M sucrose gradient in gradient buffer (10 mM HEPES, pH 7.8, 5 mM EDTA, 2 mM DTT), and centrifuged for 1 h at 117 000 × *g*_max_ at 4°C. Mitochondria were recovered from the interface, four volumes of gradient buffer were added, and mitochondria were pelleted at 10 000 × *g* for 10 min at 4°C. The sample was divided in half, and one sample was left untreated (the mitochondrial fraction) and the other treated with 0.1% (w/v) digitonin on ice for 10 min. Samples were pelleted at 15 000 × *g* for 10 min at 4°C, then divided into two again, and one sample treated with 25 μg/ml proteinase K at 37°C for 30 min. Reactions were stopped by adding PMSF to a final concentration of 5 mM, then mitochondria were pelleted again at 15 000 × *g* for 10 min at 4°C. WCE and nuclear fractions were treated with 250 U Benzonase (Sigma–Aldrich) for 30 min at 37°C to fragment genomic DNA prior to protein extraction.

### Immunofluorescence, microscopy, and image analysis

U2OS cells were plated into six-well plates on high-precision cover slips 24 h prior to induction, and then expression of TOP3A-BirA*-HA constructs was induced with 1–2.5 ng/ml doxycycline for a further 18 h. Cells were fixed using 4% (v/v) paraformaldehyde (PFA) containing 0.02% (w/v) EM-grade glutaraldehyde (G5882, Sigma) for 10 min at RT. Cells were permeabilized using 0.5% (w/v) Triton X‐100 in PBS for 15 min and blocked using 5% (w/v) BSA in PBS for 30 min followed by overnight incubation with primary antibodies (at a dilution of 1:500) in blocking solution at 4°C. After extensive washing with PBS, cover slips were incubated with secondary antibodies (at a dilution of 1:500) in blocking solution. Details of primary and secondary antibodies used are provided in Supplementary Table S1. Cover slips were subsequently washed extensively in PBS, incubated with DAPI in PBS for 5 min followed by a final PBS wash, then mounted on slides using ProLong Glass Antifade Mountant (Invitrogen, P36980) and left to cure in the dark for 24–48 h before imaging.

Airyscan images were acquired on a Zeiss LSM800 with Airyscan microscope using a 63 × 1.4 NA objective. The 405, 488, and 640 nm laser lines were used for image acquisition in conjunction with refractive index–matched immersion oil (Zeiss). Chromatic aberration was determined by imaging TetraSpeck™ microspheres (Invitrogen, T7279). Following Airyscan processing of the raw images using the Airyscan processing function in the ZEN software, chromatic aberration was corrected using the Huygens Chromatic Aberration Corrector (Scientific Volume Imaging). Pearson correlation coefficients between channels were determined for individual cells using the Huygens Colocalization Analyser Advanced (Scientific Volume Imaging) without background subtraction by manually drawing regions of interest (ROIs) for individual cells.

For presentation of representative cells, the background for each channel was determined by imaging samples without primary antibody (for TOM20) or without expression of an HA-tagged construct (for TOP3A), and image intensities were adjusted accordingly using Fiji (ImageJ).

### BioID

Expression of TOP3A-BirA*-HA and COX8-BirA*-HA (BirA* fused to the MTS of human COX8A) was induced in 245 mm × 245 mm square dishes (Thermo Fisher Scientific) with 10 ng/ml doxycycline at 70%–80% confluency. After 24 h, biotin was added to 2 μM, and after a further 24 h, cells were harvested, pelleted by centrifugation at 300 × *g* for 5 min at 4°C, and washed twice with PBS. Mitochondria were isolated as described for subcellular fractionation, omitting the sucrose gradient step.

BioID was performed following the protocol described in [29], with minor modifications. Mitochondrial pellets were lysed in 1 ml lysis buffer [50 mM Tris–HCl, pH 7.4, 500 mM NaCl, 0.2% (w/v) SDS, 1 mM DTT, and 1 × protease inhibitors] for 10 min at room temperature with rotation. Lysates were then diluted by the addition of 100 μl of 20% (v/v) Triton X-100 and 900 μl of ice-cold 50 mM Tris–HCl (pH 7.4). Cleared lysates were obtained by centrifugation at 16 500 × *g* for 10 min at 4°C.

Dynabeads MyOne Streptavidin C1 (Thermo Fisher Scientific) were pre-washed with equal volumes of lysis buffer and 50 mM Tris–HCl (pH 7.4), and 100 μl of washed beads were incubated with the cleared lysates overnight at 4°C with rotation. Beads were then washed once with 1.5 ml of 2% (w/v) SDS, followed by 1.5 ml of wash buffer [0.1% (v/v) deoxycholic acid, 1% (v/v) Triton X-100, 1 mM EDTA, pH 8.0, 50 mM HEPES pH 7.5], and then five times with 1.5 ml of 50 mM Tris–HCl (pH 7.4).

Bound proteins were eluted using 5 ng/μl trypsin, 1 mM TCEP in 50 mM Tris–HCl (pH 7.5), with incubation at room temperature for 30 min with gentle agitation. To alkylate reduced cysteines, 5 mM chloroacetamide (CAA) was added, and samples were incubated overnight at 37°C. The reaction was stopped by adding 2 μl of 50% (v/v) formic acid, and peptides were dried using vacuum centrifugation prior to LC-MS/MS analysis.

Peptides were separated on a 25 cm, 75 μm internal diameter PicoFrit analytical column (New Objective) packed with 1.9 μm ReproSil-Pur 120 C18-AQ media (Dr Maisch) using an EASY-nLC 1200 (Thermo Fisher Scientific). The column was maintained at 50°C. Buffer A and B were 0.1% (v/v) formic acid in water and 0.1% (v/v) formic acid in 80% (v/v) acetonitrile, respectively. Peptides were separated on a segmented gradient from 6% to 31% buffer B for 57 min and from 31% to 44% buffer B for 5 min at 200 nl / min. Eluting peptides were analysed on an Orbitrap Fusion Tribrid mass spectrometer (Thermo Fisher Scientific). MS1 spectra were acquired at 60 000 resolutions in the 350–1500 m/z range. Precursors with charge state from 2 to 7 only were selected for higher-energy collision dissociation (HCD) fragmentation using 27% normalized collision energy. The corresponding MS2 spectra were acquired at 30 000 resolutions with an AGC target of 2e5 and 80 maximum injection time. Upon fragmentation, precursors were put on a dynamic exclusion list for 45 s. Cycle time was set to 1 s.

### BioID protein identification and quantification

Raw data were analysed with MaxQuant version 1.6.1.0 [30] using the integrated Andromeda search engine [31]. Peptide fragmentation spectra were searched against the canonical and isoform sequences of the human reference proteome (proteome ID UP000005640, downloaded September 2018 from UniProt). Methionine oxidation and protein N-terminal acetylation were set as variable modifications; cysteine carbamidomethylation was set as a fixed modification. The digestion parameters were set to ‘specific’ and ‘Trypsin/P’. The minimum number of peptides and razor peptides for protein identification was 1; the minimum number of unique peptides was 0. Protein identification was performed at a peptide-spectrum matches and protein false discovery rate of 0.01. The ‘second peptide’ option was on. Successful identifications were transferred between the different raw files using the ‘Match between runs’ option. Label-free quantification (LFQ) [32] was performed using an LFQ minimum ratio count of 2. LFQ intensities were filtered for at least two valid values in at least one group and imputed from a normal distribution with a width of 0.3 and downshift of 1.8. Differential abundance analysis was performed using limma [33] in R.

### Protein purification

TOP3A variants carrying a C-terminal 6 × His‐tag were expressed in *Spodoptera frugiperda* Sf9 cells. Cells were harvested and lysed in 20 mM Tris–HCl (pH 8), 500 mM NaCl, 10 mM β‐mercaptoethanol, and 1 ×  protease inhibitors using one round of freeze–thaw using liquid nitrogen. Cell debris was removed by centrifugation at 75 000 × *g* for 45 min at 4°C in a Sorvall Surespin 630 rotor in a Thermo Scientific Sorvall WX 100 ultracentrifuge, and initial protein purification was performed using His‐Select Nickel Affinity Gel (Sigma–Aldrich) equilibrated with Nickel Buffer (25 mM HEPES, pH 7, 400 mM NaCl, 10% (v/v) glycerol and 10 mM β‐mercaptoethanol) containing 5 mM imidazole. The column was washed using Nickel Buffer containing 10 mM imidazole, and protein was eluted with Nickel Buffer containing 250 mM imidazole. The tagged protein was cleaved in 1 l of Nickel Buffer by dialysis with TEV protease at 4°C overnight. The cleavage product was loaded again onto His‐Select Nickel Affinity Gel, retaining the flow-through. This flow-through was subsequently purified using HiTrap Heparin HP (GE Healthcare Life Sciences) equilibrated with Buffer A (25 mM HEPES, pH 7, 200 mM NaCl, 10% (v/v) glycerol, 1 mM DTT, 0.5 mM EDTA, pH 8, and 1× protease inhibitors), and the protein was eluted as a linear gradient with Buffer B (25 mM HEPES, pH 7, 1.2 M NaCl, 10% (v/v) glycerol, 1 mM DTT, 0.5 mM EDTA, pH 8, and 1× protease inhibitors). The TOP3A peak fractions were determined using SDS–PAGE, and the protein was further purified using a Superdex 200 16/600 gel filtration column (GE Healthcare Life Sciences) equilibrated with Buffer C (25 mM HEPES, pH 7, 400 mM NaCl, 10% (v/v) glycerol, 1 mM DTT, 0.5 mM EDTA, pH 8, and 1× protease inhibitors). The resulting purified human TOP3A protein variants were concentrated using HiTrap SP HP (GE Healthcare Life Sciences) equilibrated with Buffer A and eluted with a linear gradient of Buffer B.

For purification of MPP, a 6 × His-tagged MPP construct containing Mas1 and Mas2 subunits for expression in *E. coli* was kindly provided by Johannes M. Herrmann. Four liters of *E. coli* BL21 (D3) were cultured in LB medium at 37°C to an OD_600_ of 0.7. Protein expression was induced by adding 1 mM isopropylthio-β-galactoside (IPTG), and the culture was incubated for an additional 4 h at 37°C. Cells were harvested by centrifugation.

For cell lysis, the bacterial pellet was resuspended in lysis buffer (50 mM Tris–HCl, pH 7.4, 250 mM NaCl, 2 mM β‐mercaptoethanol, 0.2% (v/v) NP-40, 1 mg/ml lysozyme, and 1× protease inhibitors) and lysed using two freeze–thaw cycles using liquid nitrogen. The lysate was then homogenized and centrifuged at 20 000 rpm for 45 min at 4°C in a Sorvall Surespin 630 rotor to remove debris. The supernatant was loaded onto a His-Select Nickel Affinity Gel (Sigma–Aldrich) pre-equilibrated with Nickel buffer (25 mM HEPES, pH 7, 400 mM NaCl, 10% glycerol, 10 mM β‐mercaptoethanol, and 5 mM imidazole). After washing with the same buffer containing 10 mM imidazole, proteins were eluted using this buffer containing 250 mM imidazole. The eluted protein was purified over HiTrap Heparin HP (GE Healthcare Life Sciences) equilibrated with 25 mM HEPES (pH 8), 200 mM NaCl, 10% glycerol, 1 mM DTT, 0.5 M EDTA (pH 8), and 1 × protease inhibitors. Elution was performed using a linear gradient of this buffer containing 1.2 M NaCl. For final purification, the elute was applied to a HiTrap MonoQ HP (GE Healthcare Life Sciences), equilibrated and eluted as earlier.

### Determination of TOP3A cleavage by tandem mass tag labelling

Flp-In T-REx 293 cells were induced to express TOP3A carrying C-terminal Strep-tag II and 3 × FLAG tags for 2 days, then cells were harvested and crude mitochondria isolated as described earlier. Affinity pulldown was performed as previously described [34], and Strep-tag II fusion proteins were eluted with D-desthiobiotin.

Cleaved TOP3A C-terminally tagged proteins (4 µg) were buffer exchanged and concentrated on 3 kDa filters (final volume of 100 µl in 50 mM TEAB). The sample was incubated with an excess of tandem mass tag reagent (TMT, 10-plex, Thermo Fisher) according to the manufacturer’s instructions, followed by evaporation of acetonitrile. TMT reacts with free amines at N-terminal peptides and lysine side chains. The labelled samples were reduced using 5 mM TCEP at 37°C for 2 h, then alkylated with 10 mM MMTS at RT for 20 min, and digested with 1 µg of trypsin (Promega) in solution at 37°C for 3 h in 0.5% sodium deoxycholate (SDC) in 50 mM TEAB buffer. The SDC was removed by acidification.

### *In vitro* cleavage of synthesized peptides using MPP

Peptide sequences are provided in Supplementary Table S2. Each peptide (5 µg) was incubated in triplicate with recombinant MPP (3.48 or 10.44 µg) in 150 mM NaCl, 10% glycerol, 50 mM Tris–HCl (pH 7.5), and either 0.1, 0.3, or 0.5 mM MnCl_2_ (as indicated) at 30°C for 1 h as described in [35]. Control reactions omitted the MPP protein. For absolute quantification of peptide cleavage, each peptide (6 µg) was incubated with recombinant MPP (or water as a control) as earlier, then the internal standard peptide was added (1:2 ratio of iSTD:peptide), and the reaction adjusted to pH 8 by the addition of TEAB to 50 mM. Cysteine residues in the peptide samples were reduced using 5 mM DTT at 60°C for 10 min and alkylated by the addition of iodoacetamine to 10 mM.

### Mass spectrometry analysis for TOP3A cleavage

Peptide samples from all experiments were purified using HiPPR Detergent Removal Resin and Pierce peptide desalting spin columns (Thermo Scientific). The peptides were dried and dissolved in 3% (v/v) ACN in 0.2% (v/v) FA prior to MS analysis. The fractions were analysed on a Q Exactive HF [for TMT labelling and parallel reaction monitoring (PRM) experiments] or an Orbitrap Fusion Lumos (for monitoring of wild-type and KGRQ peptide cleavage) mass spectrometer interfaced with Easy-nLC1200 liquid chromatography system (Thermo Fisher Scientific). Peptides were trapped on an Acclaim Pepmap 100 C18 trap column (100 μm × 2 cm, particle size 5 μm, Thermo Fisher Scientific) and separated on an in-house packed analytical column (75 μm × 30 cm, particle size 3 μm, Reprosil-Pur C18, Dr Maisch) using a gradient from 5% to 80% (v/v) acetonitrile in 0.2% (v/v) formic acid over 60 min at a flow of 300 nl/min. For the PRM method, a 30 min gradient was used.

The MS-instruments operated in data-dependent acquisition (DDA) mode. Precursor ion mass spectra were acquired at 60 000 (QEHF) or 120 000 (Lumos) resolution with m/z range 400–1600, and dynamic exclusion was set to 30 or 45 s. The 10 most intense precursor ions, charge states 2–7, were isolated with a 1.2 (QEHF) or 3 (Lumos) m/z isolation window for fragmentation with HCD at 28 NCE and detection in the Orbitrap at 30 000 resolution. PRM analyses were performed on a QEHF instrument set up as described earlier but in the PRM mode. The inclusion list contained the alkylated cleaved peptide (m/z 739.0841 Da) and the heavy isotope-labelled iSTD version (m/z 741.5861 Da) with charge state 4. The MS data were acquired with an isolation width of 2 m/z and fragments acquired with a resolution of 30 000.

### Database matching for TOP3A cleavage

Raw files were processed and analysed with Proteome Discoverer (Ver 2.2 or 3.0, Thermo Scientific). The data were matched against a custom database including the TOP3A or peptide sequences and background proteins using Mascot or Sequest as search engines with a precursor tolerance of 5 ppm (10 ppm for PRM experiments) and a fragment ion tolerance of 0.02 Da. Semitryptic peptides were accepted with one missed cleavage. Methionine oxidation was set as dynamic and cysteine alkylation as fixed. For the C-terminally tagged sample, TMT on lysine and peptide N-termini were set as a variable modification. In experiments using synthetic peptides, no enzyme was specified and without TMT modifications. All fragment spectra were manually reviewed.

In the PRM experiments, raw files were imported into Skyline (v22.2) for peak integration and quantification. The doubly charged fragment ions for quantification were *b13* to *b17*. Fragment ion chromatograms were manually reviewed. Quantification was performed using the sum of five fragments, normalized to heavy iSTDs.

### *In vitro* relaxation assays

Supercoiled pUC19 (250 ng) was incubated with the indicated quantities of recombinant TOP3A protein in 20 μl reactions that also contained 40 mM HEPES (pH 7.6), 1 mM MgCl_2_, and 30% (v/v) glycerol at 37°C for 45 min. TOP3A proteins were prepared in dilution buffer (25 mM HEPES, pH 7.6, 100 mM NaCl, 10% (v/v) glycerol, 1 mM DTT, and 100 μg/ml BSA). Positive control reactions consisted of 250 ng pUC19 incubated with 5 U of *E. coli* Topoisomerase I (New England Biolabs) in 1 × CutSmart buffer incubated as for TOP3A reactions earlier. Reactions were stopped by the addition of 1 μl of 20 mg/ml proteinase K, 2 μl of 5% (w/v) SDS, and 2 μl of 50 mM EDTA (pH 8), then incubated for a further 30 min at 37°C. Samples were separated on 0.8% (w/v) agarose gels in 1 × TAE buffer at 50 V for 3.5 h. Gels either contained 500 ng/ml ethidium bromide or were separated without ethidium bromide and then stained with 500 ng/ml ethidium bromide in water for 45 min. Gels were visualized using UV light in a Gel Doc XRS (Bio-Rad Laboratories).

### *In vitro* decatenation substrate synthesis and decatenation assays

ssDNA catenanes were synthesized as described in [23], based upon the method of [36]. Oligos R1, R2, H1, and H2 (10 pmol each) were denatured at 90°C for 3 min, then cooled at 0.1°C/s to 25°C in 17 μl reactions in 1 × T4 DNA ligase buffer (NEB). Oligos S1 and S2 (20 pmol each) were then added and incubated at 25°C for 20 min. T4 DNA ligase (400 U) was added, and the mixture incubated for 2 h at 25°C, then Proteinase K was added to a concentration of 1 mg/ml and incubated for a further 15 min at 25°C. Products were separated on denaturing PAGE gels containing 12% (v/v) acrylamide/bis (19:1), 7 M urea, and 1 × TBE, stained with 1 × SYBR Gold, then bands corresponding to ssDNA catenanes were excised using a scalpel and eluted by soaking in 1 × TBE overnight. The substrate concentration was determined by quantification of bands on a PAGE gel alongside a standard curve.

For decatenation reactions, 50 fmol of ssDNA catenane substrate was incubated with the indicated quantities of recombinant TOP3A protein [diluted in 20 mM Tris–Cl, pH 8, 200 mM NaCl, 1 mM DTT, 10% (v/v) glycerol, 0.5 mM EDTA, and 100 μg/ml BSA] in 20 μl reactions containing 25 mM Tris–HCl (pH 7.4), 5 mM MgCl_2_, 50 mM NaCl, 1 mM DTT, and 100 μg/ml BSA at 37°C for 30 min. Reaction products were separated on 12% denaturing PAGE gels as earlier, then electroblotted onto nylon membranes (Cytiva Hybond-N+) at 50 V for 1 h in 1 × TBE and crosslinked by exposure to 1200 mJ/cm^2^ UV at 254 nm. Membranes were probed overnight at 42°C using 10 pmol each of oligos R1 probe and R2 probe, which were labelled using T4 polynucleotide kinase (New England Biolabs) and 2 μl of [γ‐^32^P] ATP (10 mCi/ml, 3000 Ci/mmol, Hartmann Analytic). Following hybridization, membranes were washed for 3 × 20 min with 50 ml of 1 × saline-sodium citrate buffer containing 0.1% (w/v) SDS, and imaged using a Typhoon FLA 9500.

All oligonucleotide and probe sequences are provided in Supplementary Table S2.

### Electrophoretic mobility shift assay

A 40 nt-long ssDNA oligonucleotide (see Supplementary Table S2 for sequence) was 5′ radiolabelled with [γ-^32^P] ATP using T4 polynucleotide kinase (New England Biolabs). Reactions consisted of 10 fmol of labelled ssDNA oligonucleotide, 25 mM HEPES (pH 7.5), 100 μg/ml BSA, 1 mM DTT, 10% (v/v) glycerol, 70 mM NaCl, and the indicated amount of recombinant TOP3A in a total volume of 20 μl. Samples were incubated at 37°C for 15 min, then transferred to ice before being separated on 8% polyacrylamide gels at 150 V for 50 min in 0.5 × TBE at 4°C. Reaction products were visualized by autoradiography.

### *In vitro* assay quantification and data analysis

Images were imported into ImageLab version 6.1 (Bio-Rad Laboratories). For EMSA experiments, bands corresponding to both bound and unbound oligonucleotide were manually selected and quantified for each lane, and the percentage of bound oligonucleotide was calculated as a proportion of the total signal. For relaxation assays, bands corresponding to supercoiled substrate were quantified and expressed as a percentage of the total signal in the lane. For decatenation assays, bands corresponding to the substrate and both decatenated products were separately quantified and expressed as the proportion of the decatenated products relative to the total signal. Data were plotted using Prism (GraphPad), and two-way ANOVA was used to determine statistically significant differences between conditions. Significance values used throughout the paper are: *P < .05, **P < .01, ***P < .001, ^****^P < .0001.

## Results

### The C-terminus of mitochondrial TOP3A is truncated

**Figure 1.**
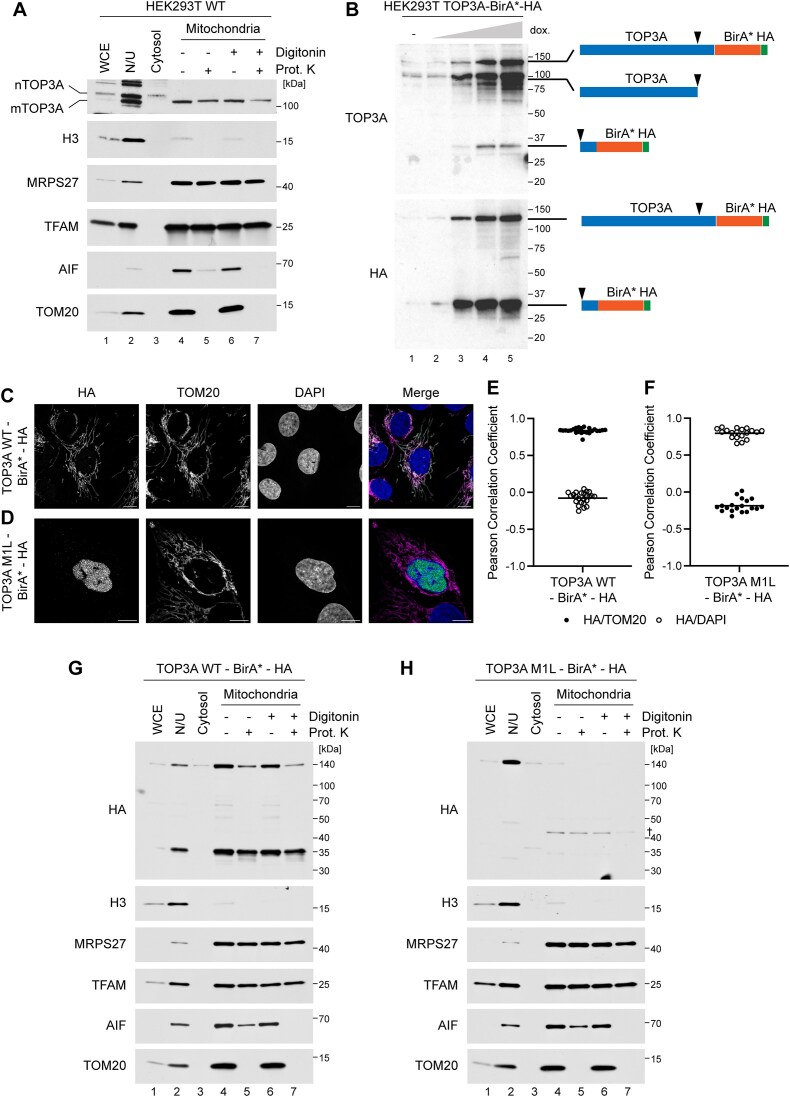
A truncated form of TOP3A is associated with mitochondria. (**A**) Subcellular localization of TOP3A variants in HEK293T cells using western blotting. Cells were fractionated using differential centrifugation into whole cell extract (WCE), nuclei and unbroken cells (N/U), cytosolic, and mitochondrial fractions. Mitochondria were additionally treated with proteinase K to degrade externally-bound proteins in the absence (lanes 4–5) or presence (lanes 6–7) of 0.1% digitonin. The migration of the nuclear and mitochondrial isoforms of TOP3A (nTOP3A and mTOP3A) is indicated. Marker proteins used are: H3 (nucleus), MRPS27 and TFAM (mitochondrial matrix), AIF (intermembrane space), and TOM20 (control for proteinase K efficacy). (**B**) Cleavage of TOP3A. Stably-transfected Flp-In T-REx 293 cells were induced to express TOP3A-WT-BirA*-HA using the indicated concentrations of doxycycline for 2 days, and lysates were analysed using western blotting using a polyclonal TOP3A antibody (top) or a HA antibody (bottom) to detect only the C-terminal fragment. Diagrams show TOP3A cleavage products, with black arrowheads indicating the expected cleavage site. (**C, D**) Localization of TOP3A variants using Airyscan confocal laser scanning microscopy. Stably-transfected U2OS cells were induced to express either TOP3A-WT-BirA*-HA (**C**) or TOP3A-M1L-BirA*-HA (**D**), and the expressed protein was detected using an anti-HA antibody. TOM20 is used as a mitochondrial marker and DAPI as a nuclear marker. The merged image shows HA in green, TOM20 in magenta, and DAPI in blue. Scale bars represent 20 μm. (**E, F**) Box plots showing the co-localization of expressed TOP3A variants (HA) with mitochondria (TOM20) or the nucleus (DAPI), expressed as Pearson correlation coefficients. Data represent values from 24 cells for TOP3A-BirA*-HA (**E**) or 20 cells for M1L-TOP3A-BirA*-HA (**F**). (**G, H**) Subcellular localization of TOP3A-WT-BirA*-HA (**G**) or TOP3A-M1L-BirA*-HA (**H**) expressed in Flp-In T-REx 293 cells using subcellular fractionation and western blotting as in panel (A). † represents residual signal from previous probing of the membrane for MRPS27.

We have previously noted that the mitochondrial isoform of TOP3A appears to have a lower molecular weight than the nuclear isoform [16]. To confirm this, we used differential centrifugation to isolate mitochondria from HEK293T cells and analysed the resulting cellular fractions using western blotting. Isolated mitochondria were additionally treated with proteinase K, either in the presence or absence of digitonin, to remove contaminating proteins from the outer surface of mitochondria. This confirmed that TOP3A appears as two bands, with the higher molecular weight band associated with fractions containing the nucleus, and the lower molecular weight band associated with the mitochondrial matrix (Fig. 1A).

We initially considered that the shorter mitochondrial isoform of TOP3A may result from cleavage of the N-terminal MTS, which would require the MTS cleavage site to lie downstream of translation start site for the nuclear isoform. However, when expressing a TOP3A fusion protein (TOP3A-BirA*-HA) to identify proximal proteins using BioID, we observed a major cleavage product containing the C-terminal domain of the fusion protein (Fig. 1B). We therefore considered that the truncated mitochondrial form of TOP3A instead results from proteolytic cleavage at the C-terminus. BioID experiments using the TOP3A-BirA*-HA fusion protein identified several factors of the mtDNA replication and mitochondrial RNA (mtRNA) metabolism machineries (Supplementary Fig. S1), likely reflecting proximity to non-cleaved TOP3A-BirA*-HA. We next expressed TOP3A-BirA*-HA, either in its wild-type form or as an M1L variant (in which the start codon required for expression of the MTS-containing isoform of TOP3A has been mutated; M1L-TOP3A-BirA*-HA) in U2OS cells. Transfection of cells with these constructs, followed by analysis of protein localization using Airyscan confocal microscopy, confirmed that the C-terminal domain of the full-length TOP3A-BirA*-HA localizes to mitochondria, whereas the M1L mutant localizes to the nucleus (Fig. 1C–F).

To further characterize the localization and cleavage of these TOP3A fusion proteins, we performed western blotting of subcellular fractions from Flp-In T-REx 293 cells stably transfected with TOP3A-BirA*-HA or M1L-TOP3A-BirA*-HA. When probing for the tagged C-terminal domain of the fusion protein (Fig. 1G), the predominant mitochondrial TOP3A-BirA*-HA signal appeared as a truncated product of ~35 kDa, representing the BirA* domain and HA tag in addition to ~9 kDa of the TOP3A C-terminus. This truncated band was not observed in cells expressing the M1L variant of the protein (Fig. 1H), confirming that C-terminal truncation of TOP3A occurs only upon its localization to the mitochondrial matrix.

### Identification of the cleavage site of mitochondrial TOP3A

**Figure 2.**
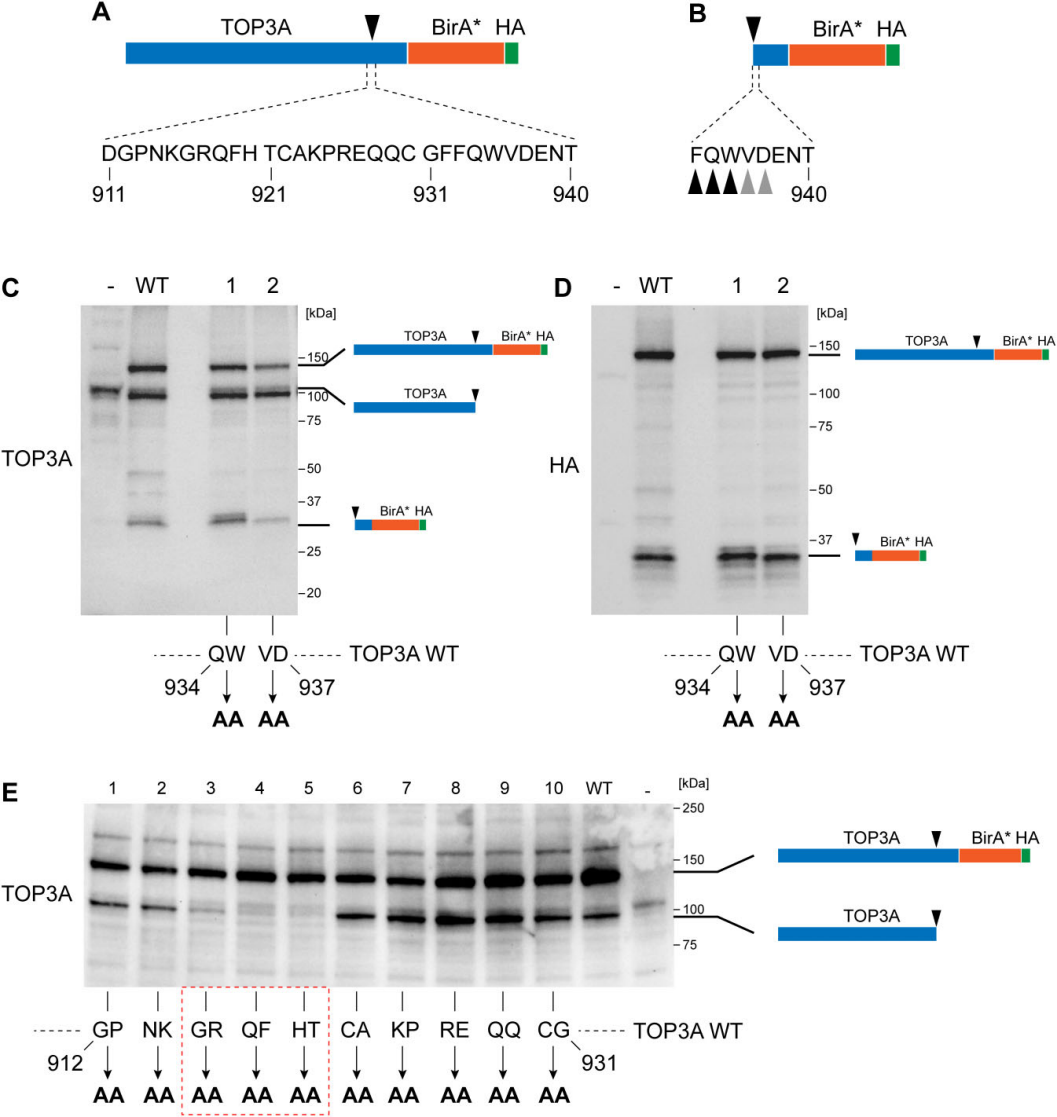
Identification of the C-terminal cleavage site of mitochondrial TOP3A. (**A**) Diagram of the structure of the TOP3A-WT-BirA*-HA construct used for identification of the C-terminal cleavage site, showing the sequence of the region identified. (**B**) Location of the termini of the TOP3A cleavage fragment. The TOP3A-WT-BirA*-HA construct was expressed in Flp-In T-REx 293 cells, then the C-terminal cleavage fragment was immunoprecipitated using anti-HA beads, and N-termini were identified using TMT labelling followed by LC–MS2 analysis. Black arrowheads indicate peptide ends identified at high abundance, and grey arrowheads indicate ends identified at lower abundance. Mass spectra for these fragments are shown in Supplementary Fig. S2. (**C, D**) Alanine scanning to identify residues critical for TOP3A cleavage. Flp-In T-REx 293 cells were induced to express either TOP3A-WT-BirA*-HA (WT) or variants in which p.934–935 (QW) or p.936–937 (VD) were substituted for alanine residues. The cleavage of these fusion proteins was assessed using western blotting with a polyclonal anti-TOP3A antibody (**C**) or an anti-HA antibody (**D**) to detect only the C-terminal fragment. (**F**) Alanine scanning of the upstream region of the TOP3A C-terminus. Stably transfected Flp-In T-REx 293 cells were induced to express wild-type TOP3A-BirA*-HA or variants representing substitution of sequential pairs of amino acids to alanine residues, spanning the region TOP3A p.912–931 (as indicated in the lower panel). Cleavage of TOP3A was determined using western blotting using a polyclonal anti-TOP3A antibody. Mutations that prevent TOP3A cleavage are highlighted with a red box. ‘-’ indicates non-transfected (parental) cells.

To identify the site of TOP3A C-terminal cleavage in mitochondria, we expressed TOP3A-BirA*-HA in Flp-In T-REx 293 cells and immunoprecipitated the C-terminal (HA-containing) proteolytic fragments. These fragments were labelled using TMT, which reacts with free amines on N-terminal peptides and lysine side chains, and analysed using liquid chromatography–mass spectrometry (LC–MS). Fragments were identified by comparing their experimental MS2 fragmentation spectra to theoretical spectra from a database, matching observed fragment ions to predicted patterns based on peptide sequences and TMT modification. Peptide spectra matches (PSMs) are instances where MS2 spectra are assigned to a same fragment and can give indications about the absolute amounts of that fragment. This identified the most abundant peptides as having N-termini at TOP3A p.933–935, and less abundant fragments with N-termini at p.936–937 (Fig. 2A and B and Supplementary Fig. S2). To determine whether these sites represented the true proteolytic cleavage sites, we expressed TOP3A-BirA*-HA variants with alanine substitutions at positions p.934–935 or p.936–937. Analysis of these proteins using western blotting found that these variants did not impair cleavage of the TOP3A C-terminus (Fig. 2C and D). We therefore considered that the TOP3A sites identified using LC–MS may represent the products of partial N-terminal degradation of the cleaved protein fragment and focused on the region immediately upstream of these sites. By constructing a series of TOP3A variants in which successive pairs of codon positions between TOP3A p.912 and p.931 were mutated to alanine residues, we found that C-terminal cleavage of TOP3A was abolished by mutation of positions p.914–921 (Fig. 2E).

**Figure 3.**
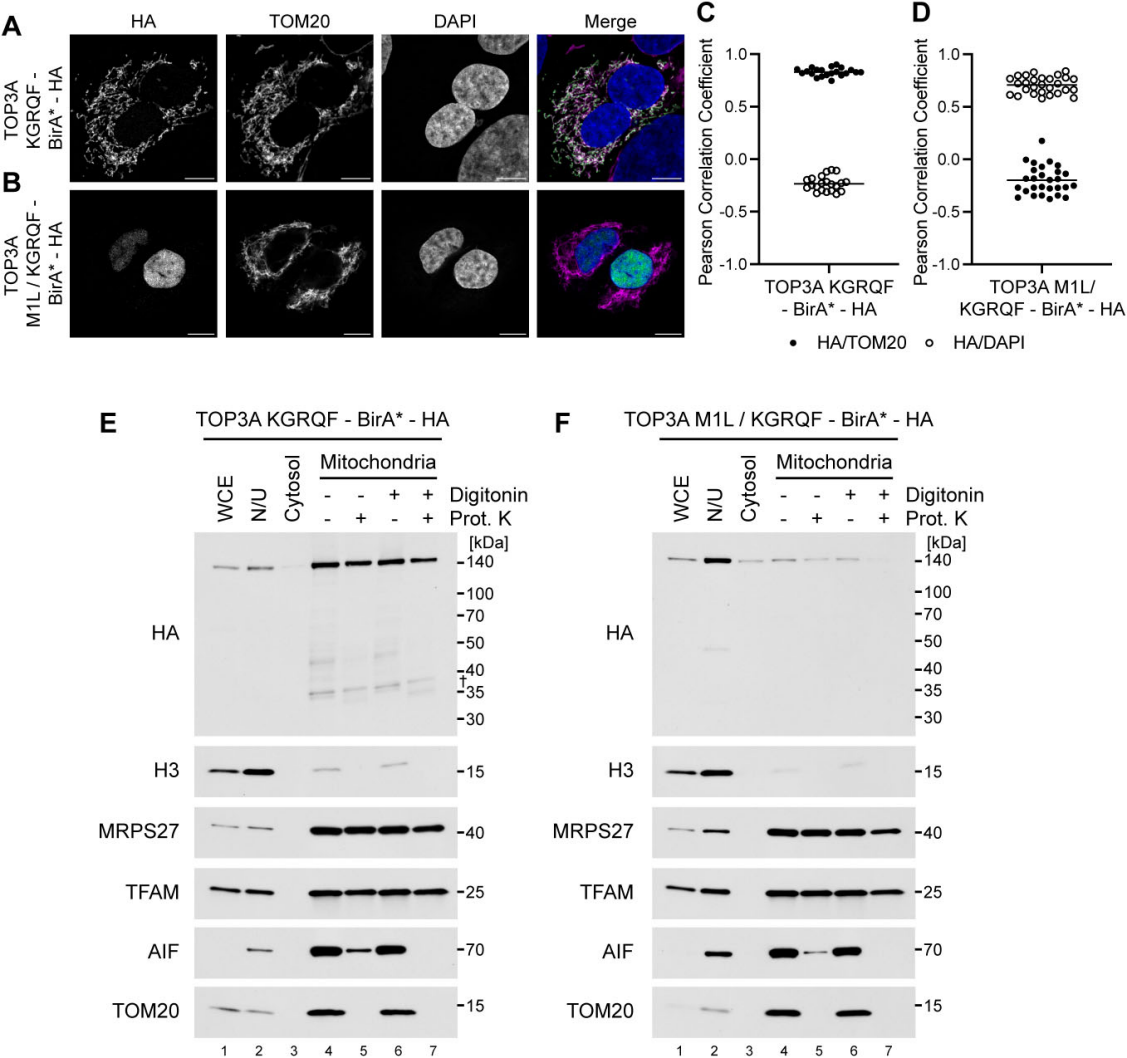
Amino acids 915–919 are crucial for TOP3A C-terminal cleavage. (**A, B**) Localization of TOP3A variants using Airyscan confocal laser scanning microscopy. Stably transfected U2OS cells were induced using doxycycline to express TOP3A following mutation of residues 915–919 to alanine (TOP3A-KGRQF-BirA*-HA, **A**) or an equivalent construct with the additional mutation of M1, required for mitochondrial localization (TOP3A-M1L/KGRQF-BirA*-HA, **B**) and detected using a HA-specific antibody. TOM20 was used as a mitochondrial marker and DAPI as a nuclear marker. The merged images show HA in green, TOM20 in magenta, and DAPI in blue. Scale bars represent 20 μm. (**C, D**) Box plots showing the co-localization of expressed TOP3A (HA) variants with mitochondria (TOM20) or the nucleus (DAPI) as in panels (A) and (B), expressed as Pearson correlation coefficients. Data represent values from 21 cells for TOP3A-KGRQF-BirA*-HA (**C**) or 28 cells for TOP3A-M1L/KGRQF-BirA*-HA (**D**). (**E, F**) Subcellular localization of TOP3A-KGRQF-BirA*-HA (**E**) or TOP3A-M1L/KGRQF-BirA*-HA (**F**) expressed in stably-transfected Flp-In T-REx 293 cells using subcellular fractionation and western blotting. The C-terminus of TOP3A is detected using a HA antibody. Marker proteins used are H3 (nucleus), MRPS27 and TFAM (mitochondrial matrix), AIF (intermembrane space), and TOM20 (control for proteinase K efficacy). WCE, whole cell extract; N/U, nuclei/unbroken cells. † represents residual signal from previous probing of the membrane for MRPS27.

To pinpoint the critical residues required for cleavage, we created a variant of TOP3A in which each residue of the KGRQF motif (p.915–919) is replaced by an alanine residue (TOP3A-KGRQF-BirA*-HA). Imaging of U2OS cells transfected with this construct confirmed that the TOP3A-KGRQF-BirA*-HA variant retains its mitochondrial localization, while additionally mutating the M1 initiation codon (TOP3A-M1L/KGRQF-BirA*-HA) abolishes mitochondrial localization and results in localization of the protein to the nucleus (Fig. 3A–D).

Fractionation of these cell lines using differential centrifugation and analysis of the resulting cell fractions using western blotting demonstrated that mutation of the KGRQF motif effectively prevents C-terminal cleavage of TOP3A (Fig. 3E). As expected, no cleavage of TOP3A was observed in cells where the M1 initiation codon was additionally mutated (Fig. 3F).

In parallel experiments, we mutated only the first four amino acids of this motif (KGRQ) and performed localization experiments using microscopy and cell fractionation. As expected, the TOP3A-KGRQ-BirA*-HA variant localized to mitochondria, while additional mutation of M1 (TOP3A-M1L/KGRQ-BirA*-HA) prevented its mitochondrial localization (Supplementary Fig. S3A–D). Analysis of TOP3A cleavage using cell fractionation indicated that mutation of KGRQ impaired, but not abolished, C-terminal cleavage of TOP3A (Supplementary Fig. S3E and F). These experiments indicate that cleavage occurs near amino acid position p.915–919 of TOP3A, with F919 being critical for this processing.

### The C-terminus of mitochondrial TOP3A is cleaved by MPP

**Figure 4.**
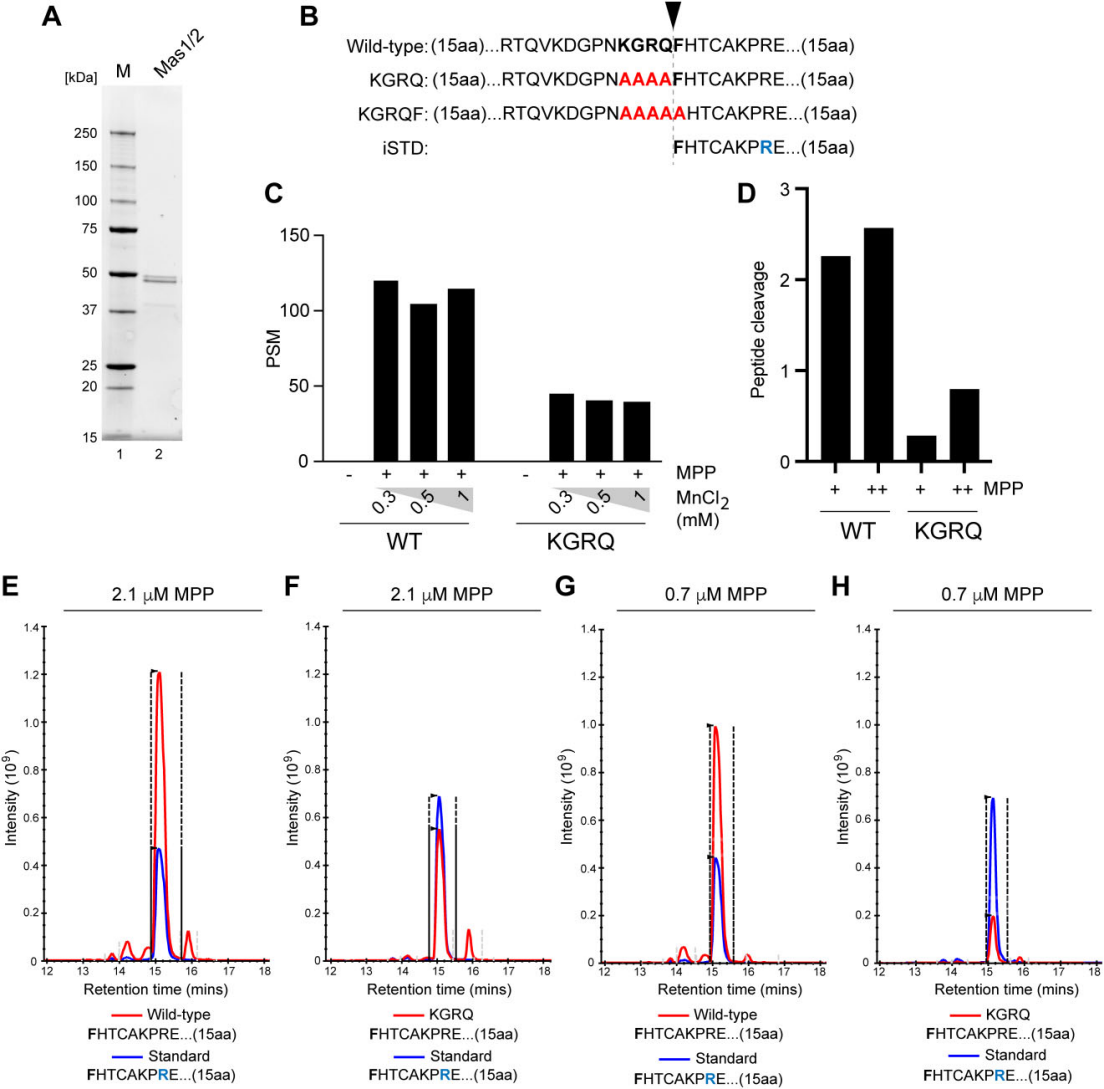
The C-terminus of mitochondrial TOP3A is cleaved by MPP. (**A**) Purification of MPP. The purified Mas1/2 complex (2.4 pmol) was separated by 4%–20% SDS–PAGE and visualized using stain-free imaging. (**B**) Diagram of synthetic peptides used for characterization of the TOP3A C-terminal cleavage reaction using mass spectrometry. Wild-type, KGRQ, and KGRQF peptides correspond to TOP3A p.891–942, and the iSTD peptide corresponds to TOP3A p.919–942. Amino acid substitutions are shown in red, and isotopically labelled arginine (^13^C6^15^N4) in the iSTD peptide is shown in blue. (**C**) PSM corresponding to cleavage between TOP3A p.918 and p.919. Wild-type or KGRQ peptides were incubated in the absence or presence of recombinant MPP at the indicated concentrations of MnCl_2_, and the resulting samples were analysed using DDA tandem mass spectrometry for identification of peptide fragments. (**D**) Quantification of TOP3A cleavage by MPP as in panels (E–H), represented as the ratio between the TOP3A peptide cleavage product and the iSTD peptide. (E–H) Absolute quantitative analysis of TOP3A cleavage by MPP using PRM. Wild-type (**E + G**) or KGRQ (**F + H**) peptides were incubated with 2.1 μM (**E, F**) or 0.7 μM (**G, H**) recombinant MPP. The resulting cleavage product between TOP3A p.918 and p.919 (red line) was quantified relative to the iSTD peptide (blue line) carrying an isotopically labelled arginine residue.

We next sought to define the enzymatic activity responsible for C-terminal cleavage of TOP3A, and to reconstitute this cleavage reaction. Although many proteases are known to localize to mitochondria, many either specifically degrade damaged or misfolded proteins or process protein termini in a way that is incompatible with the observed cleavage of TOP3A [37]. We therefore considered that TOP3A may be cleaved at its C-terminus by the MPP during protein import into mitochondria. Although MPP typically removes N-terminal MTSs, it is also capable of cleaving proteins internally into separate functional domains [35, 38]. To test this, we expressed and purified the yeast MPP homologue, consisting of the two subunits Mas1 and Mas2, using bacterial cells (Fig. 4A). We then designed three peptides corresponding to the C-terminal sequence of TOP3A; one wild-type and two variants in which the KGRQ or KGRQF (p.915–919) residues were substituted with alanine (Fig. 4B). These peptides were incubated with recombinant MPP, and the resulting peptide fragments identified using mass spectrometry. Following incubation of wild-type TOP3A peptide with MPP, the most abundant peptide product represented cleavage before F919 (Fig. 4C), matching the site seen to be essential for TOP3A cleavage in cultured cells (Fig. 3). Substitution of the KGRQ sequence to alanine residues greatly reduced cleavage at this site (Fig. 4C), similar to the effect observed in cell culture experiments. To quantitatively analyse the cleavage of these peptides by MPP, we used PRM to measure the generation of the cleavage product relative to a spiked iSTD of identical sequence but containing an isotopically labelled arginine residue (^13^C6^15^N4). The wild-type TOP3A sequence was again efficiently cleaved before F919, and this cleavage was impaired by substitution of the KGRQ motif to AAAA (Fig. 4D–H). Similar results were observed using two different concentrations of MPP (2.1 µM, Fig. 4E and F, and 0.7 µM, Fig. 4G and H). We additionally used a peptide representing substitution of the KGRQF sequence to alanine (TOP3A-KGRQF). Since the alteration of F919 prevented quantification of peptide cleavage relative to the iSTD peptide, samples were also analysed in DDA mode without targeting specific peptides. This analysis found that substitution of F919 resulted in a markedly lower number of PSMs corresponding to cleavage at this site (Supplementary Table S3), further supporting the conclusion that F919 is essential for TOP3A cleavage by MPP.

### C-terminal cleavage modulates the activity of TOP3A

**Figure 5.**
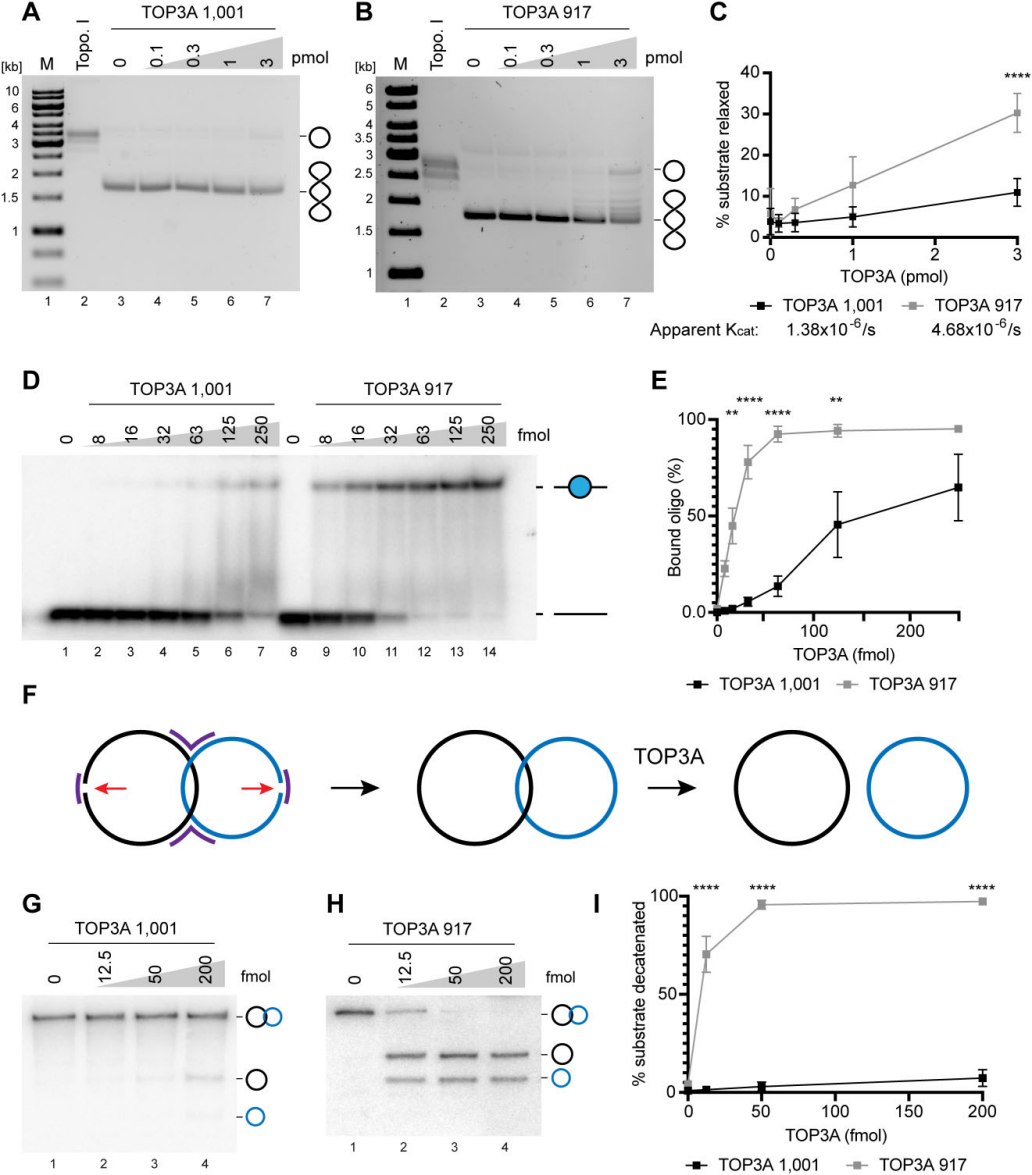
C-terminal cleavage stimulates the activity of mitochondrial TOP3A. (A, B) Relaxation of negatively supercoiled plasmid DNA by full-length TOP3A (TOP3A 1 001) and truncated TOP3A (TOP3A 917) isoforms. pUC19 DNA was incubated with the indicated concentrations of recombinant TOP3A 1 001 (**A**) or TOP3A 917 (**B**), then separated on 0.8% agarose gels and stained with ethidium bromide. ‘M’ (lane 1) indicates marker, and ‘Topo. I’ (lane 2) represents incubation of pUC19 DNA with *E. coli* Topoisomerase I as a positive control. (**C**) Quantification of plasmid relaxation assays as in panels (A) and (B). Data represent mean values from three independent experiments; error bars represent SEM (two-way ANOVA with Šidák’s multiple comparisons test, ^****^P < .0001). (**D**) EMSA to assess the ssDNA binding of TOP3A variants. A 40 nt ssDNA oligonucleotide (10 fmol) was incubated with the indicated quantities of TOP3A 1 001 or TOP3A 917, then separated by 8% denaturing PAGE and imaged using autoradiography. The migration of unbound and bound oligonucleotide is indicated. (**E**) Quantification of DNA binding as in panel (D). Data represent mean values from three independent experiments; error bars represent SEM (two-way ANOVA with Šidák’s multiple comparisons test, **P < .01, ^****^P < .0001). (**F**) Construction of singly linked catenated ssDNA substrates for analysis of TOP3A decatenation activity. Two ssDNA oligonucleotides (black and blue) are held in place by short oligonucleotides (purple) and ligated together (red arrows). Incubation of purified substrate with recombinant TOP3A results in the formation of separate circular oligonucleotides. ssDNA decatenation activity of TOP3A 1 001 (**G**) and TOP3A 917 (**H**) determined using singly linked ssDNA catenanes as in panel (F). The migration of catenated substrate and decatenated circular ssDNA products is indicated. (**I**) Quantification of ssDNA decatenation activity of TOP3A 1001 and TOP3A 917 as in panels (G) and (H). Data represent mean values from three independent experiments; error bars represent SEM (two-way ANOVA with Šidák’s multiple comparisons test, ^****^P < .0001).

Although the function of the C-terminal region of TOP3A is poorly understood, it is known that this domain is not essential for catalytic activity [39]. We therefore considered that proteolytic cleavage of the TOP3A C-terminus may alter its activity within mitochondria. To address this question, we expressed and purified full-length (TOP3A 1001) and truncated (TOP3A 917) forms of TOP3A using insect cells (Supplementary Fig. S4A). We first assessed the ability of these protein variants to relax a negatively supercoiled pUC19 plasmid, finding that the truncated isoform of TOP3A had a moderately higher activity than the full-length form (Fig. 5A–C). Reaction rates were used to calculate the apparent k_cat_ for the two isoforms (Fig. 5C). Similar experiments in which reaction products were separated on gels containing ethidium bromide, which promotes the supercoiling of covalently closed plasmid molecules, showed that the truncated form of TOP3A also shows some nicking activity (Supplementary Fig. S4B and C). We next assessed the ability of the two TOP3A isoforms to bind to a 40 nt ssDNA oligonucleotide using an electrophoretic mobility shift assay (EMSA), finding that TOP3A 917 had a higher DNA-binding activity than the full-length protein (Fig. 5D and E). Since TOP3A has an essential role in the decatenation of mtDNA replication products [16], we sought to determine the ability of the two TOP3A isoforms to decatenate model DNA substrates. We constructed singly linked ssDNA catenanes using oligonucleotides of different lengths, which can be decatenated by recombinant TOP3A and detected using Southern blotting [23, 36] (Fig. 5F). The full-length isoform of TOP3A was able to partially decatenate this substrate, while the truncated TOP3A isoform was able to fully decatenate the substrate, and at lower protein concentrations (Fig. 5G–I). This greater decatenation activity of the truncated TOP3A isoform was not dependent upon the exact location of the C-terminal end of TOP3A, as a slightly longer version of the protein (TOP3A 934, corresponding to the downstream cleavage site identified in Fig. 2A) possessed an almost identical decatenation activity to TOP3A 917 (Supplementary Fig. S4D and E). Together, these experiments indicate that the cleaved mitochondrial form of TOP3A exhibits significantly enhanced catalytic activity compared with the full-length protein.

## Discussion

We show that TOP3A undergoes proteolytic cleavage by MPP within mitochondria, removing ~90 amino acids from its C-terminus and significantly altering the enzyme’s biochemical properties. Although little is known about the biological function of the C-terminal domain of TOP3A, we suggest that the removal of this region could influence the biology of TOP3A in several ways.

The removal of the C-terminus appears to strongly promote the ability of TOP3A to decatenate ssDNA substrates. The nuclear isoform of TOP3A is part of a complex together with the OB-fold proteins RMI1 and RMI2, and the helicase BLM [40–43]. RMI1 binds directly to TOP3A in the nucleus and has been shown to stimulate its ssDNA decatenation activity by stabilizing a more open structural form of the enzyme [16, 39, 44]. Given that the mitochondrial isoform of TOP3A has an essential role in the decatenation of mtDNA replication products, it was surprising that RMI1 does not appear to also localize to mitochondria [16]. However, our current results suggest the possibility that the truncation of the TOP3A C-terminus, and the higher decatenation activity associated with this variant, may compensate for the lack of RMI1 within mitochondria. In this scenario, the binding of RMI1 to nuclear TOP3A may constitute a form of regulation of TOP3A activity that is not required within mitochondria.

AlphaFold predictions of the full-length TOP3A structure (not shown) highlight the intrinsic disorder of the C-terminal tail and suggest that this region may mediate transient or context-dependent interactions rather than forming a stable structural element. Future high-resolution structural approaches such as cryo-EM will be essential to determine whether the C-terminal 90 amino acids of TOP3A engage in regulatory interactions with DNA or with partner proteins.

The C-terminus of TOP3A also contains a predicted nuclear localization sequence (p. 961–984, prediction score of 10.0 using cNLS Mapper [45]), which is within the region that is proteolytically removed from the mitochondrial isoform of TOP3A. Once within mitochondria, this sequence would not be expected to function, as nuclear-encoded mitochondrial proteins such as TOP3A are post-translationally imported into mitochondria as linear polypeptides before folding into their mature forms and remaining in the mitochondrial matrix. Thus, removal of the C-terminal region may also serve to prevent nuclear re-import of the mitochondrial isoform, reinforcing its compartmental restriction and functional specialization.

Our recent results have found that neither of the human TOP2 isoforms localize to human mitochondria or contribute to maintenance or expression of mtDNA, and that mitochondria possess only two topoisomerase activities, TOP3A and TOP1MT [15]. This implies that the decatenation activity of TOP3A is essential within mitochondria, as this activity cannot be compensated for by TOP1MT, and appears to be in agreement with the observed potent decatenation activity of the truncated mitochondrial TOP3A variant. The ability of TOP3A to relax negatively supercoiled DNA, on the other hand, would be redundant with TOP1MT, in agreement with the observation that loss of both TOP3A and TOP1MT causes a synergistic defect in mtDNA replication and expression [15].

A recent report has modelled the effect of a Bloom syndrome-causing TOP3A truncating mutation in mice. This variant removes ~240 amino acids from the TOP3A C-terminus and results in impaired activity of the TOP3A–RMI1–RMI2 complex [46]. This suggests that further truncation of the protein beyond the C-terminus of the mitochondrial isoform is detrimental to its function, or possibly that the C-terminal region is required for the stimulation of TOP3A activity by RMI1–RMI2.

A precedent for functionally significant processing by MPP is found in *Saccharomyces cerevisiae*, where the Arg5,6 polyprotein is cleaved at both the N-terminus and an internal site to generate two separate enzymes involved in arginine biosynthesis [38]. This demonstrates that MPP can act beyond simple presequence removal, actively diversifying protein function. The C-terminal cleavage of mitochondrial TOP3A described here similarly produces an isoform with distinct biochemical activity, enhancing its ability to decatenate ssDNA. While this processing is likely a constitutive maturation step that tailors the enzyme for its role in mtDNA maintenance, it remains possible that cleavage efficiency is regulated under certain physiological conditions.

Future studies could concentrate upon the interaction between TOP3A and RMI1 within the nucleus, and whether this constrains or directs the activity of TOP3A towards specific substrates. More broadly, our results also raise the possibility that post-translational protein cleavage by MPP could constitute a mechanism to also modulate the activity or function of other mitochondrial proteins.

## Supplementary Material

gkaf1140_Supplemental_Files

## Data Availability

Proteomics data have been deposited to the ProteomeXchange Consortium (http://proteomecentral.proteomexchange.org) via the PRIDE partner repository [47] with identifiers PXD063285 and PXD064926. Raw microscopy and blotting data have been uploaded to Zenodo and are available at doi.org/10.5281/zenodo.16872498.
