# Supplementary material for: Proteolytic cleavage activates the mitochondrial isoform of TOP3A
Source: Nucleic Acids Res. 2025 Nov 4;53(20):gkaf1140. doi: 10.1093/nar/gkaf1140 (PMC12585908; doi:10.1093/nar/gkaf1140)
Supplement: gkaf1140_Supplemental_Files [file gkaf1140_supplemental_files.zip › 180925_Erdinc_NAR_compiled_supplementary_data.pdf]

Supplementary data for:

Proteolytic Cleavage Activates the Mitochondrial Isoform of TOP3A

Direnis Erdinc[1], Christin A. Albus[2], Alejandro Rodríguez-Luis[2], Katja E. Menger[2], Annika Thorsell[3], Ilian Atanassov[4], Urška Rovšnik[1], Maria Falkenberg[1], Claes M. Gustafsson[1,*], Thomas J. J. Nicholls[2,*]

[1]Department of Medical Biochemistry and Cell Biology, University of Gothenburg, SE-405 30 Gothenburg, Sweden.

[2]Biosciences Institute, Faculty of Medical Sciences, Newcastle University, Newcastle upon Tyne, NE2 4HH, UK.

[3]Proteomics Core Facility at Sahlgrenska Academy, University of Gothenburg, SE-405 30 Gothenburg, Sweden.

[4]Proteomics Core Facility, Max-Planck-Institute for Biology of Ageing, 50931 Cologne, Germany.

*Correspondence to: thomas.nicholls@newcastle.ac.uk or claes.gustafsson@medkem.gu.se

Contents:

Supplementary Figure 1: Identification of TOP3A-proximal proteins using BioID

Supplementary Figure 2: Mass spectra of TOP3A C-terminal cleavage fragments

Supplementary Figure 3: Characterisation of TOP3A-KGRQ

Supplementary Figure 4: *In vitro* assays of TOP3A function

Supplementary Table 1: Antibodies used in this study

Supplementary Table 2: Sequences of oligonucleotides and peptides used in this study

Supplementary Figure 1

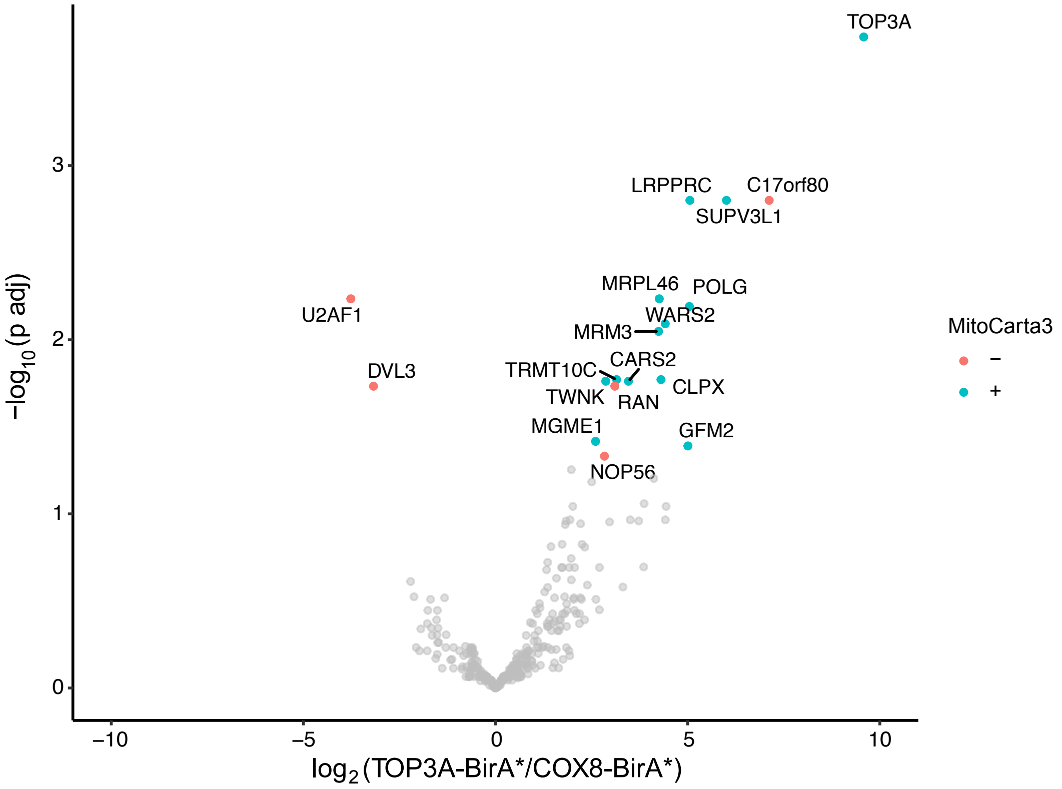

Supplementary Figure 1

Identification of TOP3A-proximal proteins using BioID. Volcano plot showing differentially enriched proteins following expression of TOP3A-BirA*-HA relative to a mitochondrially-targeted BirA* protein (COX8-BirA*). Biotinylated proteins were isolated using streptavidin beads and identified using mass spectrometry. Significant hits (Student's *t*-test, Benjamini-Hochberg false discovery rate correction $p<0.05$, $\log2FC>\pm2$) are labelled and colour coded according to their presence (blue) or absence (red) from MitoCarta 3.0.

Supplementary Figure 2

A

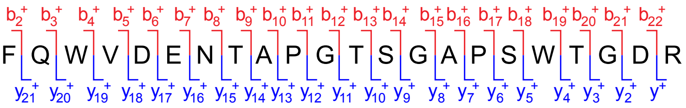

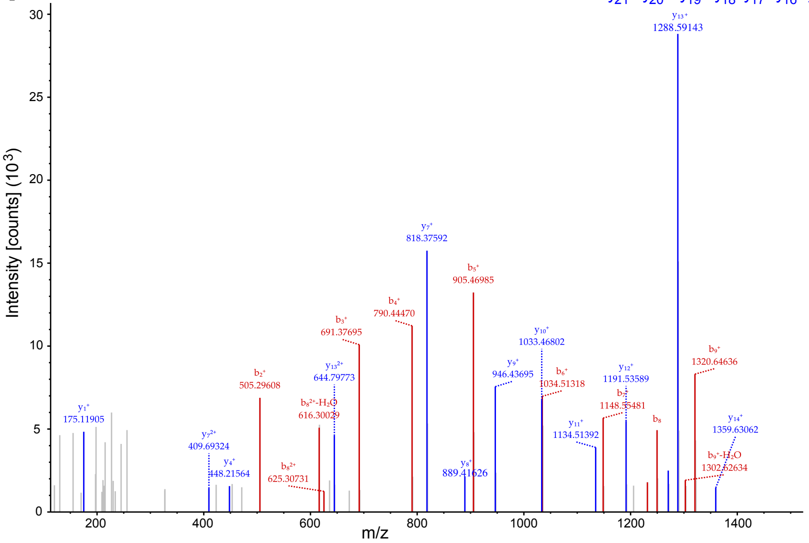

#1	b+	b2+	seq.	y+	y2+	#2
1	377.23862	189.12295	F-TMT6plex			22
2	505.29720	253.15224	Q	2231.99521	1116.50125	21
3	691.37651	346.19189	W	2103.93664	1052.47196	20
4	790.44493	395.72610	V	1917.85732	959.43230	19
5	905.47187	453.23957	D	1818.78891	909.89809	18
6	1034.51446	517.76087	E	1703.76197	852.38462	17
7	1148.55739	574.78233	N	1574.71937	787.86333	16
8	1249.60507	625.30617	T	1460.67645	730.84186	15
9	1320.64218	660.82473	A	1359.62877	680.31802	14
10	1417.69495	709.35111	P	1288.59165	644.79947	13
11	1474.71641	737.86184	G	1191.53889	596.27308	12
12	1575.76409	788.38568	T	1134.51743	567.76235	11
13	1662.79612	831.90170	S	1033.46975	517.23851	10
14	1719.81758	860.41243	G	946.43772	473.72250	9
15	1790.85469	895.93099	A	889.41626	445.21177	8
16	1887.90746	944.45737	P	818.37914	409.69321	7
17	1974.93949	987.97338	S	721.32638	361.16683	6
18	2161.01880	1081.01304	W	634.29435	317.65081	5
19	2262.06648	1131.53688	T	448.21504	224.61116	4
20	2319.08794	1160.04761	G	347.16736	174.08732	3
21	2434.11488	1217.56108	D	290.14590	145.57659	2
22			R	175.11895	88.06311	1

B

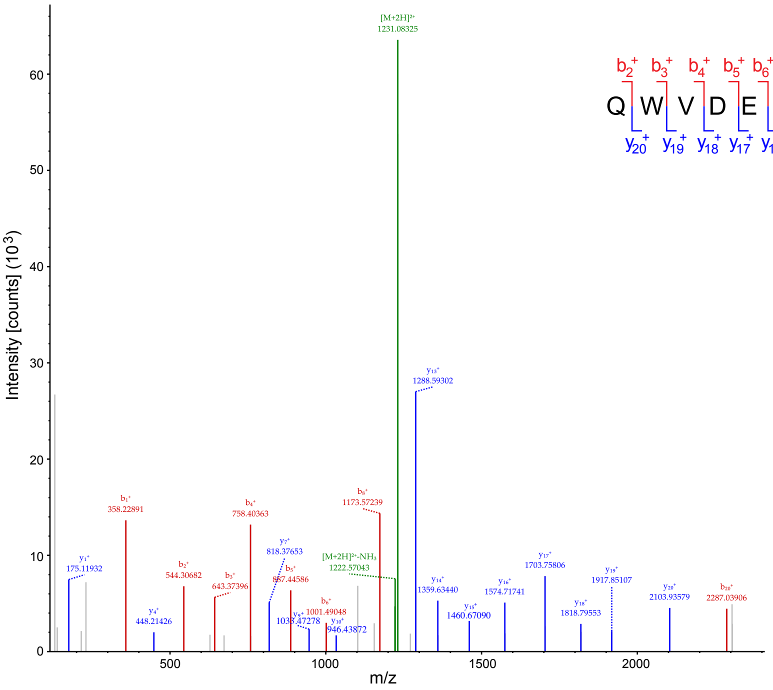

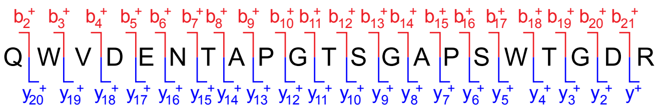

#1	b+	b2+	seq.	y+	y2+	#2
1	358.22879	179.61803	Q-TMT6plex			21
2	544.30810	272.65769	W	2103.93664	1052.47196	20
3	643.37651	322.19189	V	1917.85732	959.43230	19
4	758.40346	379.70537	D	1818.78891	909.89809	18
5	887.44605	444.22666	E	1703.76197	852.38462	17
6	1001.48898	501.24813	N	1574.71937	787.86333	16
7	1102.53665	551.77197	T	1460.67645	730.84186	15
8	1173.57377	587.29052	A	1359.62877	680.31802	14
9	1270.62653	635.81690	P	1288.59165	644.79947	13
10	1327.64800	664.32764	G	1191.53889	596.27308	12
11	1428.69567	714.85148	T	1134.51743	567.76235	11
12	1515.72770	758.36749	S	1033.46975	517.23851	10
13	1572.74917	786.87822	G	946.43772	473.72250	9
14	1643.78628	822.39678	A	889.41626	445.21177	8
15	1740.83904	870.92316	P	818.37914	409.69321	7
16	1827.87107	914.43917	S	721.32638	361.16683	6
17	2013.95039	1007.47883	W	634.29435	317.65081	5
18	2114.99806	1058.00267	T	448.21504	224.61116	4
19	2172.01953	1086.51340	G	347.16736	174.08732	3
20	2287.04647	1144.02687	D	290.14590	145.57659	2
21			R	175.11895	88.06311	1

C

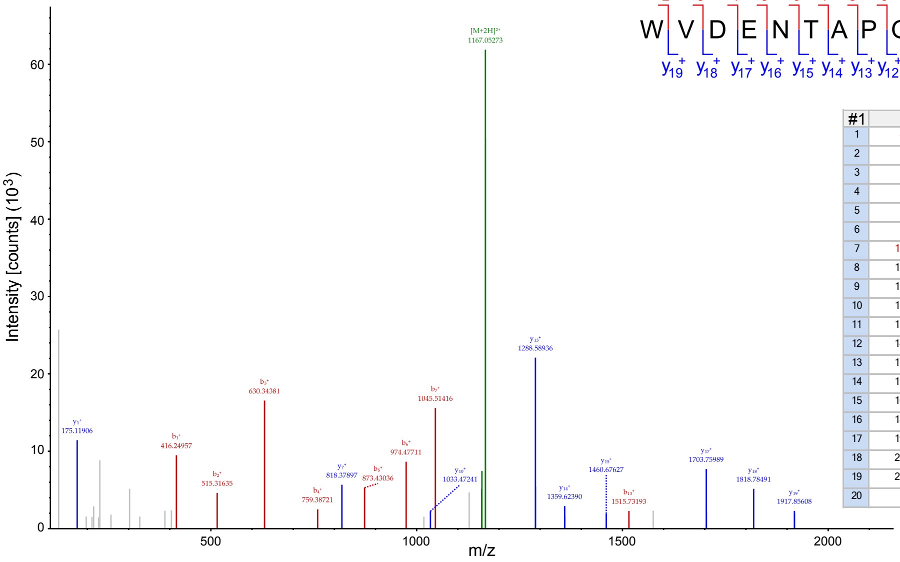

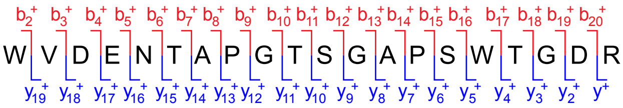

#1	b+	b2+	seq.	y+	y2+	#2
1	416.24952	208.62840	W-TMT6plex			20
2	515.31794	258.16261	V	1917.85732	959.43230	19
3	630.34488	315.67608	D	1818.78891	909.89809	18
4	759.38747	380.19737	E	1703.76197	852.38462	17
5	873.43040	437.21884	N	1574.71937	787.86333	16
6	974.47808	487.74268	T	1460.67645	730.84186	15
7	1045.51519	523.26123	A	1359.62877	680.31802	14
8	1142.56795	571.78762	P	1288.59165	644.79947	13
9	1199.58942	600.29835	G	1191.53889	596.27308	12
10	1300.63710	650.82219	T	1134.51743	567.76235	11
11	1387.66913	694.33820	S	1033.46975	517.23851	10
12	1444.69059	722.84893	G	946.43772	473.72250	9
13	1515.72770	758.36749	A	889.41626	445.21177	8
14	1612.78047	806.89387	P	818.37914	409.69321	7
15	1699.81249	850.40989	S	721.32638	361.16683	6
16	1885.89181	943.44954	W	634.29435	317.65081	5
17	1986.93949	993.97338	T	448.21504	224.61116	4
18	2043.96095	1022.48411	G	347.16736	174.08732	3
19	2158.98789	1079.99758	D	290.14590	145.57659	2
20			R	175.11895	88.06311	1

Supplementary Figure 2

Mass spectra of TOP3A C-terminal cleavage fragments. TOP3A-WT-BirA*-HA was expressed in Flp-In T-REx 293 cells, and the C-terminal fragment was immunoprecipitated, labelled using TMT and analysed using LC-MS. Spectra are shown for the major identified fragments with N-termini at F933 **(A)**, Q934 **(B)** and W935 **(C)**. Peaks are coloured to show b-ions in red and y-ions in blue. Tables show the expected fragment masses, and identified fragments are indicated in colour.

Supplementary Figure 3

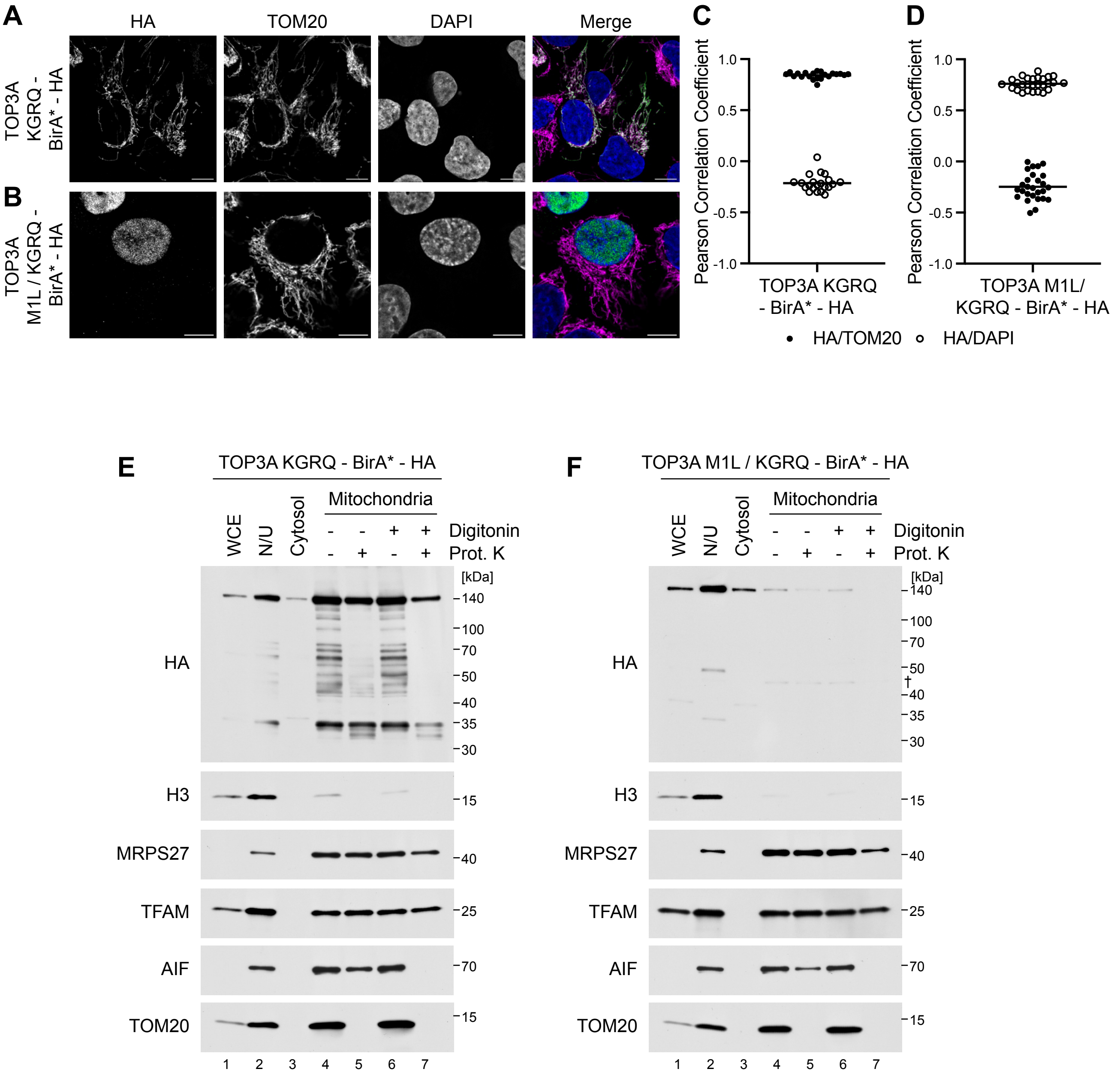

Supplementary Figure 3

(A-B) Localisation of TOP3A variants with substitution of KGRQ (TOP3A p.915-918) to alanine (TOP3A-KGRQ-BirA*-HA, **A**), or with additional mutation of M1, required for mitochondrial localisation (TOP3A-M1L/KGRQ-BirA*-HA, **B**), assessed using Airyscan confocal laser scanning microscopy. Expression of TOP3A variants was induced in stably-transfected U2OS cells using doxycycline, and detected using an HA-specific antibody. TOM20 is used as a mitochondrial marker and DAPI as a nuclear marker. The merged image shows HA in green, TOM20 in magenta, and DAPI in blue. Scale bars represent 20 µm.

(C-D) Box plots showing the co-localisation of expressed TOP3A variants (HA) with mitochondria (TOM20) or the nucleus (DAPI), expressed as Pearson correlation coefficients. Data represents values from 21 cells for TOP3A-KGRQ-BirA*-HA **(C)** or 27 cells for TOP3A-M1L/KGRQ-BirA*-HA **(D)**.

(E-F) Localisation of TOP3A-KGRQ-BirA*-HA **(E)** or TOP3A-M1L/KGRQ-BirA*-HA **(F)** expressed in stably-transfected Flp-In T-REx 293 cells, assessed using subcellular fractionation and western blotting. The C-terminus of TOP3A is detected using an anti-HA antibody. Marker proteins used are H3 (nucleus), MRPS27 and TFAM (mitochondrial matrix), AIF (intermembrane space) and TOM20 (control for proteinase K efficacy). Proteinase K-sensitive bands of intermediate size represent incomplete synthesis products that are not fully imported into the mitochondrial matrix. WCE, whole cell extract; N/U, nuclei/unbroken cells. † represents residual signal from previous probing of the membrane for MRPS27.

Supplementary Figure 4

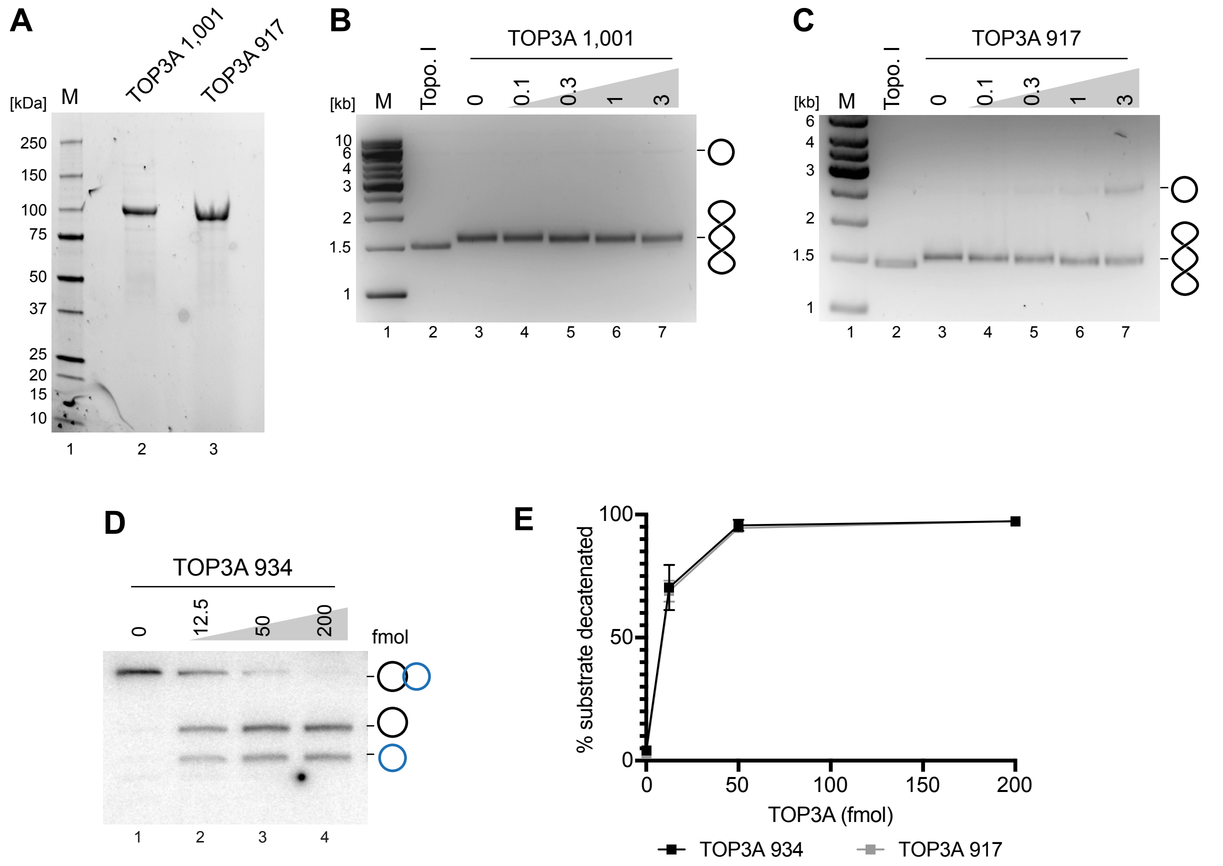

Supplementary Figure 4

(A) Purification of TOP3A variants. Recombinant TOP3A (1.8 pmol), representing the full-length protein (TOP3A 1,001) or truncated form (TOP3A 917) was separated by 4-20% SDS-PAGE and visualised using stain-free imaging. 'M' indicates marker.

(B-C) Products of pUC19 relaxation assays using TOP3A 1,001 **(B)** or TOP3A 917 **(C)** separated on ethidium-bromide-containing gels. Reactions were carried out as in Figure 5A-B, then reaction products were separated on 0.8% agarose gels containing 500 ng/ml ethidium bromide, and visualised using UV. 'M' (lane 1) indicates marker, and 'Topo. I' (lane 2) represents incubation of pUC19 DNA with *E. coli* Topoisomerase I as a positive control. The migration of nicked products (circles) and supercoiled molecules is indicated.

(D) ssDNA decatenation activity of the truncated TOP3A 934 variant, which is 17 amino acids longer at the C-terminus than the TOP3A 917 variant used in other experiments. A singly-linked ssDNA catenated substrate was incubated with the indicated quantities of recombinant TOP3A 934, and reaction products were separated by denaturing PAGE and detected using Southern blotting. The migration of catenated substrate and decatenated circular ssDNA products is indicated.

(E) Quantification of the decatenation activity of TOP3A 934 as in (D). The quantification of the decatenation activity of TOP3A 917 is reproduced from Figure 5I for comparison. Data represents mean values from three independent experiments, and error bars represent SEM.

Supplementary Table 1: Antibodies used in this study

**Primary antibodies**			
**Target**	**Supplier**	**Cat. no.**	**RRID**
AIF	Cell Signaling Technology	4642	RRID:AB_2224542
β-actin	Abcam	ab6276	RRID:AB_2223210
H3	Abcam	ab1791	RRID:AB_302613
HA	Sigma-Aldrich	H6908	RRID:AB_260070
MRPS27	Proteintech	17280-1-AP	RRID:AB_2180510
TFAM	Abcam	ab119684	RRID:AB_10900340
TOM20 (WB)	Abcam	ab78547	RRID:AB_2043078
TOM20 (IF)	Abcam	ab56783	RRID:AB_945896
TOP3A	Proteintech	14525-1-AP	RRID:AB_2205881
**Secondary antibodies**			
**Target**	**Supplier**	**Cat. no.**	**RRID**
Goat anti-rabbit IgG Alexa Fluor 488	Invitrogen	A-11008	RRID:AB_143165
Goat anti-mouse STAR RED	Abberior	STRED-1001	RRID:AB_3068620
Rabbit anti-mouse HRP	Agilent	P0260	RRID:AB_2636929
Swine anti-rabbit HRP	Agilent	P0217	RRID:AB_2728719

Supplementary Table 2: Sequences of oligonucleotides and peptides used in this study

**Oligonucleotides for EMSA**	
**Name**	**Sequence (5' - 3')**
EMSA substrate	TGTTATTATTATGTCCTACAAGCATTAATTAATTAACACA
**Oligonucleotides for Lk1 catenane synthesis**	
**Name**	**Sequence (5' - 3')**
R1	p-GACTAGAGCAGATATAATACGACTCACTATAGGGATACA ATAGGCAGCTGGACGTGTACCAAGTTAGCAGTCATG
R2	p-ATGTCTGGTTCGTCTCACGACTCATCACGCCCTATAGTG GATCAGCACACATCATATCACAGC
H1	GTACACGTCCAGCTGCCTATTGTATCGTGATGAGTCGTGA GACGAA
H2	GTGCTGATCAGTCGTATTA
S1	CTAGTCCATGAC
S2	AGACATGCTGTG
R1 probe	TCTGCTCTAGTCCATGACTGCTAAC
R2 probe	CGAACCAGACATGCTGTGATATGAT
**qPCR primers and probes**	
**Name**	**Sequence (5' - 3')**
ND1 forward	CCCTAAAACCCGCCACATCT
ND1 reverse	GAGCGATGGTGAGAGCTAAGGT
ND1 probe	VIC-CCATCACCCTCTACATCACCGCCC-MGB
B2M forward	CCAGCAGAGAATGGAAAGTCAA
B2M reverse	TCTCTCTCCATTCTTCAGTAAGTCAACT
B2M probe	6-FAM-ATGTGTCTGGGTTTCATCCATCCGACA-MGB
**Peptides**	
**Name**	**Sequence** (amino acid substitutions are shown in red, and isotopically-labelled arginine ($^{13}C6^{15}N4$) is shown in blue)
Wild-type	SGSGTSCLCSQPSVTRTVQKDGPN**KGRQFH**TCAKPREQQ CGFFQWVDENTAP
KGRQ	SGSGTSCLCSQPSVTRTVQKDGPN**AAAAFH**TCAKPREQQC GFFQWVDENTAP
KGRQF	SGSGTSCLCSQPSVTRTVQKDGPN**AAAAAH**TCAKPREQQ CGFFQWVDENTAP
Internal standard (iSTD)	FHTCAKP**R**EQQCGFFQWVDENTAP
